# Fluorescence Lifetimes of NIR-Emitting Molecules with Excited-State Intramolecular Proton Transfer

**DOI:** 10.3390/molecules28010125

**Published:** 2022-12-23

**Authors:** Yonghao Li, Dipendra Dahal, Yi Pang

**Affiliations:** Department of Chemistry, University of Akron, Akron, OH 44325, USA

**Keywords:** cyanine, ESIPT, ICT, lifetime, local excited state, charge transfer state

## Abstract

Molecular probes based on the excited-state intramolecular proton-transfer (ESIPT) mechanism have emerged to be attractive candidates for various applications. Although the steady-state fluorescence mechanisms of these ESIPT-based probes have been reported extensively, less information is available about the fluorescence lifetime characteristics of newly developed NIR-emitting dyes. In this study, four NIR-emitting ESIPT dyes with different cyanine terminal groups were investigated to evaluate their fluorescence lifetime characteristics in a polar aprotic solvent such as CH_2_Cl_2_. By using the *time-correlated single-photon counting (TCSPC)* method, these ESIPT-based dyes revealed a two-component exponential decay (τ_1_ and τ_2_) in about 2–4 nanoseconds (ns). These two components could be related to the excited *keto* tautomers. With the aid of model compounds (**5** and **6**) and low-temperature fluorescence spectroscopy (at −189 ℃), this study identified the intramolecular charge transfer (ICT) as one of the major factors that influenced the τ values. The results of this study also revealed that both fluorescence lifetimes and fractional contributions of each component were significantly affected by the probe structures.

## 1. Introduction

As one of the intrinsic properties of a fluorophore, fluorescence lifetime describes the characteristic time that a molecule remains in the excited state before returning to its ground state. Fluorescence lifetime is not dependent on the excitation methods (such as the wavelength of excitation, one or multiphoton excitation). Additionally, this photophysical property is basically not affected by the fluorescence intensity or fluorophore concentration. Since the excited state is a highly energetic state, its decay to its ground state is affected by both internal (e.g., fluorophore structure) and external conditions (e.g., solvent polarity [1] and the presence of fluorescence quencher). Due to the reasons mentioned above, fluorescence lifetime becomes a separate but complementary method to fluorescence intensity-based measurement [2].

**Figure 1:**
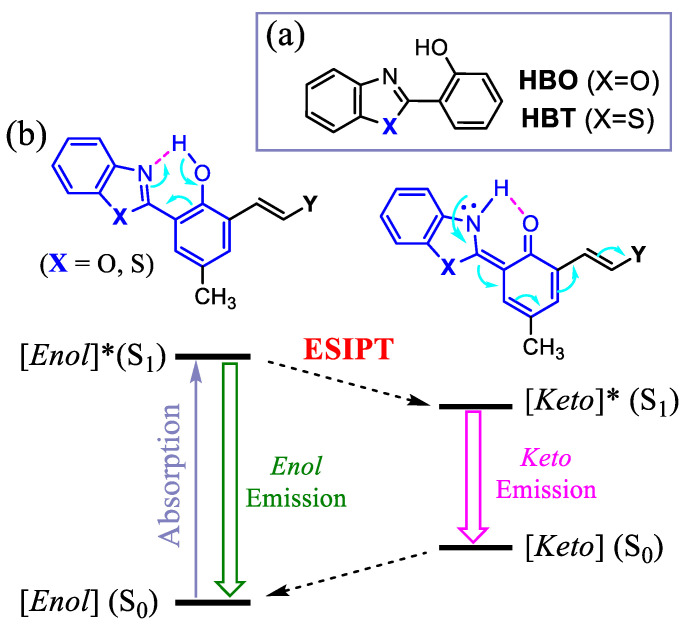
(**a**) Chemical structure of HBO and HBT. (**b**) Schematic illustration of the excited-state intramolecular proton transfer (ESIPT) process, where the asterisk indicates the excited state. After deactivation of the keto excited state (K*), a ground state retro proton transfer occurs to regenerate the enol form. In the illustration, the substituent “**Y**” represents an organic group, such as a pyridinium group in probe **1**.

Recently, excited-state intramolecular proton transfer (ESIPT) has emerged to be an attractive mechanism for the design of molecular probes, due to their unique fluorescent properties that include large Stokes shifts and dual emissions (arising from the *enol* and *keto* tautomers). The most common ESIPT fluorophores are derivatives of 2-(2′-hydroxyphenyl)benzoxazole (HBO) and 2-(2′-hydroxyphenyl) benzothiazole (HBT) (Figure 1). In previous studies, great efforts have been made to utilize the *enol* and *keto* emission, as well as their ratio, for a variety of chemical sensor applications [3]. There are also significant efforts to tune the ESIPT emission toward the near-infrared (NIR) region for improved imaging applications [4]. Most of the reported studies are relying on fluorescence intensity-based measurement, by using analyte-induced changes in fluorescence wavelengths (or color) and quantum yields.

Significant interests exist in studying the decay process of the excited ESIPT fluorophores by using time-resolved spectroscopic methods [5]. For example, HBO has been reported to exhibit a short lifetime (τ ≈ 20 ps) and a long lifetime (τ ≈ 295 ps) in hexane, with the long lifetime species being associated with its *keto* tautomer [6]. Additionally, the fluorescence of HBT is reported to exhibit one lifetime that is highly dependent on the solvent polarity, showing τ ≈ 12–17 ps in CH_3_CN and τ ≈ 100 ps in cyclohexane [7]. By using ultrafast infrared spectroscopy to monitor the process, the observed lifetime is identified to be associated with the *keto* tautomer [7]. As new ESIPT-based fluorescent probes have been developed, it is important to continue the evaluation of the fluorescence lifetime characteristics of these new materials and to learn how the structural variation could affect the lifetime parameters.

**Scheme 1:**
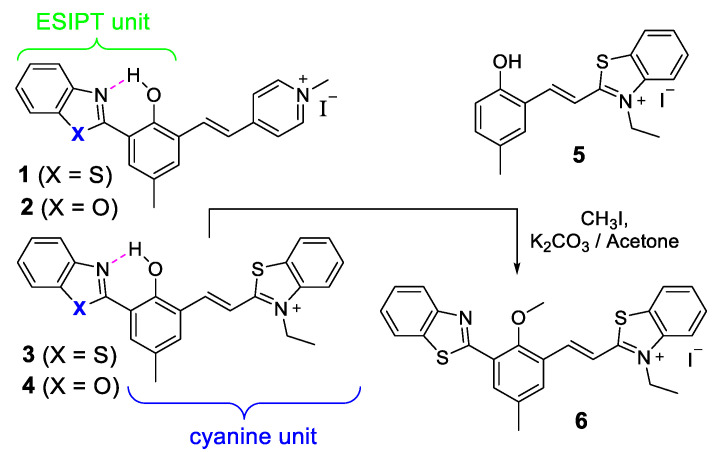
Chemical structures of probes **1**–**4**, and the model compounds **5** and **6**.

In an effort to tune the emission toward a longer wavelength, we have reported probes **1**–**4** [8,9,10]. For example, probe 1 has been shown to be a useful fluorescent dye for imaging intracellular mitochondria, cellular membranes and neuromast organs on zebrafish [9,11]. As a consequence of effective proton transfer, the dye gives only keto emission with a large Stokes shift. This is in sharp contrast to simple HBT, whose emission from enol/keto tautomers is quite sensitive to solvents [1]. As a unique structural feature, dyes **1**–**4** incorporate a styryl group with a positively charged cyanine segment as indicated by the “cyanine unit” in Scheme 1, which enhances the intramolecular charge transfer (ICT) interaction in their excited states. A recent study investigated the coupling of ESIPT and ICT of **3** by using ultrafast transient absorption spectroscopy and quantum chemical calculations, showing an ultrafast proton transfer that is associated with solvation (~1.5 picoseconds) and conformation relaxation (~13 picoseconds) [12]. As a consequence of only keto emission, these dyes exhibit a clear “transparent window” between their absorption and emission, such as dye **1** which reveals a transparent window between 500–580 nm (Supporting Information, Figures S11–S13). This is in sharp contrast to classical organic fluorescent dyes, such as fluorescein, whose fluorescence spectra always have some spectral overlap with their absorption. The lack of emission from the enol tautomer indicated that the proton transfer happens effectively in the excited states. A fundamental question is why the keto emission occurs nearly exclusively in these compounds. In order to shed some light on this fundamental process, we decide to evaluate/understand the fluorescence lifetime characteristics of compounds **1**–**4**, which have not been investigated in previous studies.

## 2. Results and Discussion

### 2.1. Synthesis

**Figure 2:**
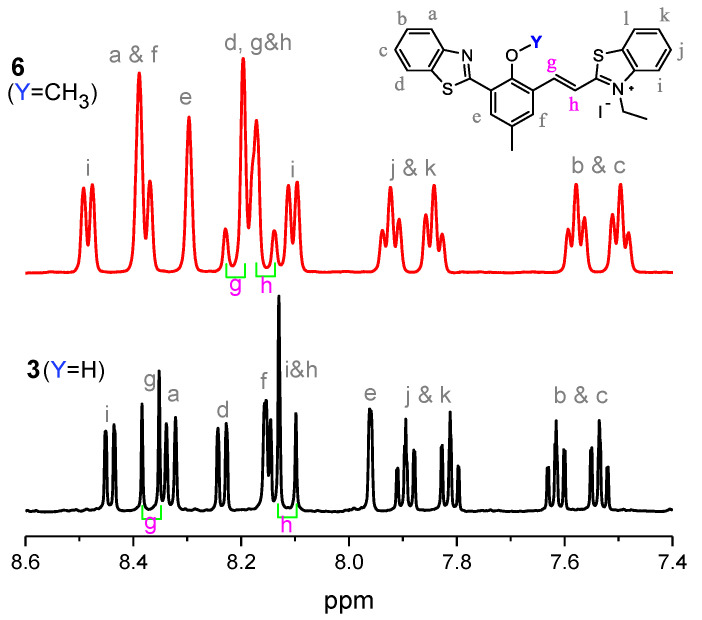
^1^H NMR spectra of **3** and **6** in DMSO-d_6_, in which the *J* coupling patterns of the vinyl protons are indicated. The alkyl protons are not shown for clarity.

Compounds **1**–**5** were synthesized by using procedures as described in our previous reports. In order to aid the study, model compound **6** was synthesized by reacting **3** with excess methyl iodide in the presence of a base at room temperature. All products are characterized by ^1^H-NMR, ^13^C-NMR and MS spectra (ESI Figures S1–S10). Different from **3**, the hydroxy group in **6** is protected, which eliminates the proton transfer later. ^1^H NMR spectrum of **6** revealed only one resonance Ar-OCH_3_ signal at ~3.93 ppm, whose integration matched well with the signals from the -CH_2_CH_3_ group (ESI Figure S8). A large coupling constant (*J* = 15 Hz) was observed from the vinyl protons in both spectra of **3** and **6** (Figure 2), showing the *trans*-CH=CH linkage. Since only *trans*-CH=CH was detected in both compounds, the reaction sequence from **3** to **6** did not have any impact on the stereochemistry of the vinyl bond linkage.

### 2.2. Spectroscopic Studies

**Figure 3:**
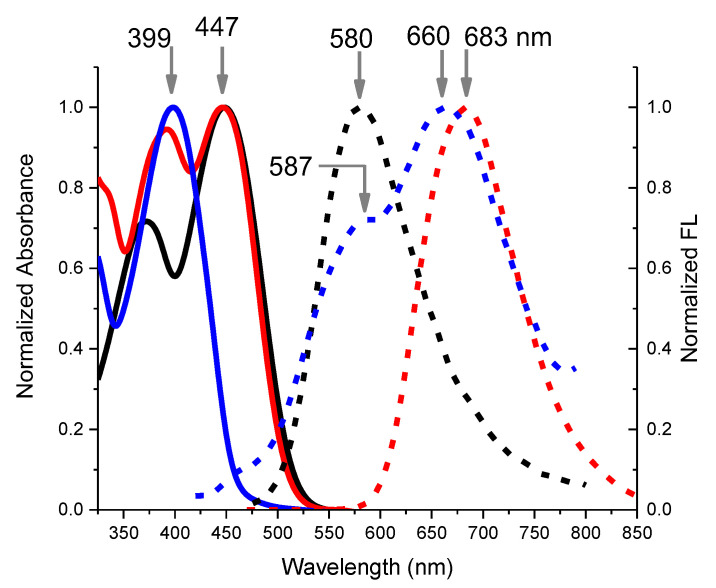
UV–vis absorption (solid line) and emission spectra (broken line) of compound **3** (red), **5** (black) and **6** (blue) in CH_2_Cl_2_ (10 μM). The excitation wavelengths were 447 nm for **3** and **5**, and 399 nm for **6**, respectively; fluorescence spectroscopy (FL).

The photophysical properties are listed in Table 1 (ESI Figures S11–S28). It was reported that both compound **3** and its effective chromophore **5** exhibited nearly identical absorption (e.g., ~447 in CH_2_Cl_2_) (Figure 3). However, the quantum yield of **3** was significantly higher than **5**, due to the coupling with the ESIPT unit in the former [8]. The structural analogues, such as **1**&**4**, also revealed similar appealing properties (i.e., high ϕ_fl_ and large Stokes shifts of these ESIPT cyanine dyes) [9]. The role of the ESIPT unit in enhancing the fluorescence was further demonstrated by the synthesis of model compound **6**, whose ϕ_fl_ was much lower than its parent compound **3**. Clearly, the intramolecular hydrogen bonding played an important role in maintaining the high fluorescence of this class of NIR-emitting dyes, partly due to increased molecular rigidity via hydrogen bonding.

**Scheme 2:**
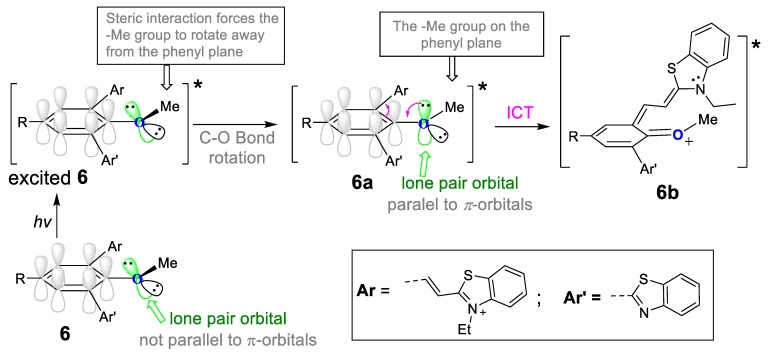
Schematic illustration of molecular geometry in ground state of **6**, and structural change from the locally excited state of 6 to the excited species **6a** and **6b**; intramolecular charge transfer (ICT).

It should be noted that the absorption of **6** (λ_max_ ≈ 399 nm) was blue-shifted from **5** by about 37–48 nm. It appeared that the introduced methyl-protecting group on the phenol perturbed the alignment of the lone pair on the oxygen atom (with the π-bond on the aromatic ring), resulting in a hypochromic shift. The observed hypochromic shift could be attributed to the steric interaction of the methoxy group. In the ground state, the methyl group would be forced to rotate away from the plane of the connected phenyl ring, due to the steric interaction with the adjacent aromatic group (**6** in Scheme 2). As a consequence, the lone pair electron on the oxygen atom (with the sp^2^ hybridization) would be away significantly from the desirable parallel position to the π-orbitals of the phenyl ring. This could prevent effective electron delocalization from the methoxy group to the phenyl ring, thereby leading to the hypochromic shift in UV–vis absorption. Free rotation of the methoxy group, relative to the connected aromatic ring, appeared to be a major factor that led to a large decrease in the fluorescence quantum yield of **6** (by a factor of ~100 in comparison with that of **3**).

Interestingly, compound **6** gave two emission peaks, with one at ~587 nm and the other at ~660 nm (Figure 3). The emission at ~587 nm matched the emission peak of **5**, which could be attributed to the effective chromophore (or cyanine subunit). It was assumed that the methyl group could adopt a coplanar position with the phenyl ring in the excited state as shown in **6a**, in which the lone pair orbital on the oxygen is aligned parallel to the π-orbitals for extended conjugation/interaction. Nearly identical emission between **6** (λ_em_ ≈ 587 nm) and **5** (λ_em_ ≈ 580 nm) supports the assumption, as the lone pair orbital on the phenol of **5** is expected to be parallel with the π-orbitals on the phenyl ring. The second emission from **6** (λ_em_ ≈ 660 nm) could be attributed to the excited species **6b**, which has lower energy than **6a** after undergoing the intramolecular charge transfer (ICT).

#### Low-Temperature Fluorescence

**Figure 4:**
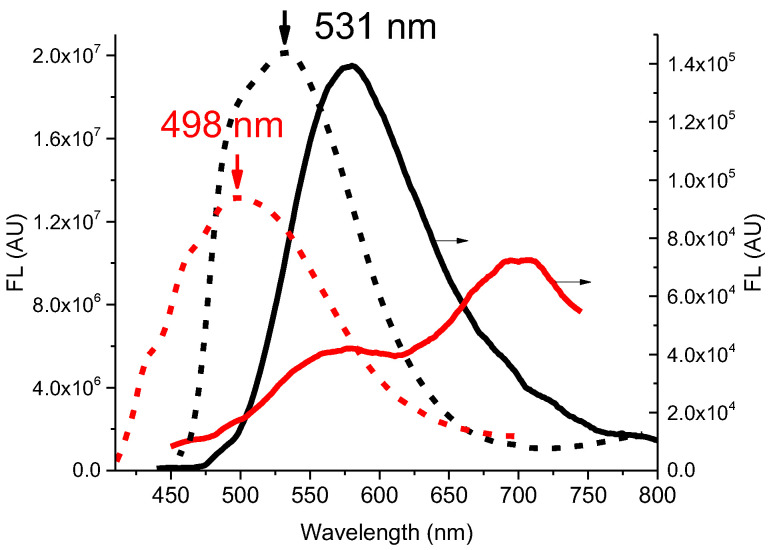
Emission spectra of 10 μM methanol solution of **5** (black) and **6** (red) at room temperature (20 °C, solid line) and in liquid nitrogen (−189 °C, broken line).

In order to explore the ICT nature of the fluorophores, we decided to acquire the fluorescence spectra at low temperatures. In the previous studies on cyanine **1** & **4** [4], [9] the molecular motion and bond changes associated with the ICT process are shown to be frozen at the low temperature, while the ESIPT process could still operate. Thus, the methanol solution (10 μM) of model compounds **5** and **6** in quartz tubes was quickly cooled by immersing the sample into liquid nitrogen in a quartz Dewar. The fluorescence spectra at low temperature (−189 °C) were significantly blue-shifted from that at room temperature 20 °C (Figure 4), as the ICT interaction was removed in the excited states. The emission of **5** was blue shifted from 581 nm to 531 nm when the temperature was decreased to −189 °C. At room temperature, the solution of **6** exhibited two emission peaks in methanol (λ_em_ ≈ 568 & 700 nm, Figure 4), similar to that in CH_2_Cl_2_ (Figure 3). Interestingly, only one emission peak was observed at −189 °C from **6**. This observation pointed to the low energy emission peak (λ_em_ ≈ 700 nm) from **6** was largely associated with ICT. In other words, the ICT interaction could play a more important role in **6**. The charge transfer accounts for the degrees of the redshift of the emission spectra from different extents of ICT nature [13]. Since the ICT was disabled when the sample was frozen, the excited **6** could not generate **6a** & **6b**, and the emission could only occur from **6**. The emission of **6** at ~700 nm at room temperature was not associated with the aggregation, as aggregation content would increase with decreasing temperature [14]. In summary, the low-temperature fluorescence supported the proposed fluorescence process in Scheme 2. 

### 2.3. Fluorescence Lifetime 

When the fluorescence decay involves multicomponents [15], the fluorescence signal decay I(t) is assumed to be the sum of individual single exponential decay from each component:

$$I\left(t\right)=\sum_{i=1}^{n}\alpha_{i}e^{-\frac{t}{\tau_{i}}}$$

In the above equation, $\tau_{i}$ are the individual lifetimes of each component, $\alpha_{i}$ represent the amplitudes of the components at t = 0, and *n* is the number of decay times. In a mixture of fluorophores, the decay times $\tau_{i}$ may be assigned to each excited species. The relative contributions of each component i are calculated from the pre-exponential values $\alpha_{i}$ weighted by the lifetime $\tau_{i}$ and given as a percentage, with $\overset{n}{\underset{i=1}{\sum }}f_{i}=1$.

$$f_{i}=\frac{\int_{0}^{\infty }I_{i}\left(t\right)dt}{\int_{0}^{\infty }I\left(t\right)dt}=\frac{\alpha_{i}\tau_{i}}{\sum_{j}\alpha_{j}\tau_{j}}$$

**Figure 5:**
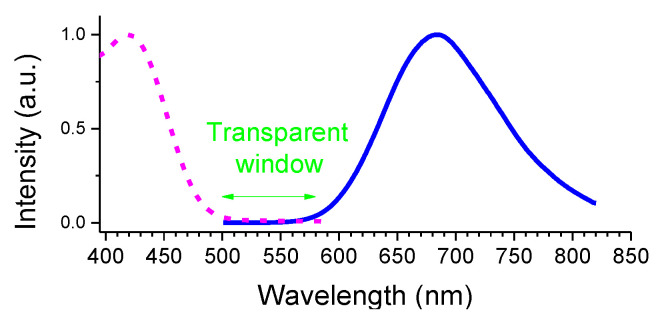
UV–vis absorption (dotted line) and fluorescence spectra (solid line) of fluorescent dye **1** (in CH_2_Cl_2_) with a large Stokes shift. The dye **1** exhibits “a transparent optical window” between 500 and 580 nm.

Our initial study was carried out in CH_2_Cl_2_ (a polar aprotic solvent), in order to avoid the dissociation of phenolic protons while providing good solubility. The steady-state fluorescence of compound **1** in CH_2_Cl_2_ at room temperature is shown in Figure 5. On the basis of a large Stokes shift, the emission peak at λ_em_ ≈ 685 nm was attributed to the *keto* emission. The absence of the absorption and emission signals between 500 and 580 nm led to an optically transparent window, suggesting that the *enol* emission was either absent or at a very low concentration.

**Figure 6:**
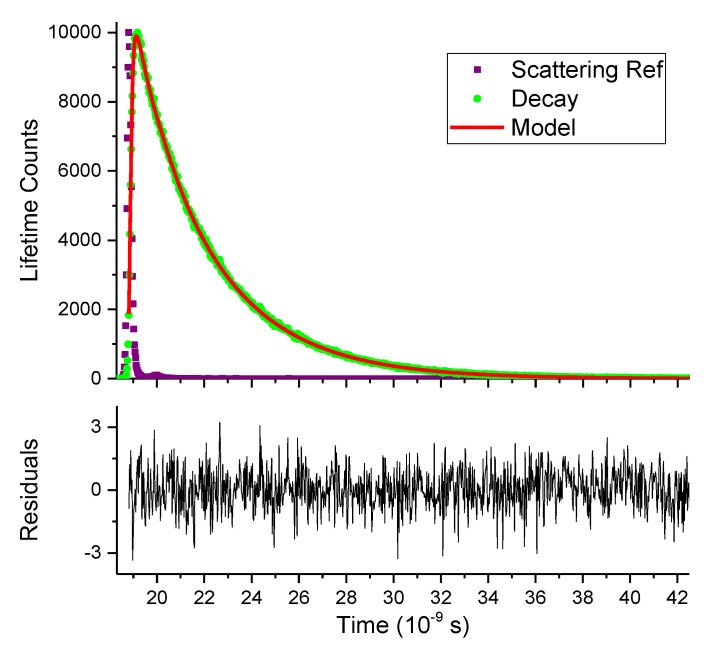
Data obtained by using the *time-correlated single-photon counting* (TCSPC) method. Sample: solution of dye **1** in CH_2_Cl_2_ (under Ar). Excitation wavelength: 405 nm (using picosecond diode Lasers). Data were collected by using a 550 nm long path filter. Reference: scattering solution. Result: τ_1_= 1.05 ns, τ_2_= 3.38 ns; $\chi_{r}^{2}$ = 1.02.

Interestingly, fluorescence lifetime measurement of **1** revealed a two-exponential decay, with τ_1_ = 1.05 ns (5.8%) and τ_2_ = 3.38 ns (94.2%), based on the best curve fitting of reduced chi-square ($\chi_{r}^{2}=1.02)$ shown in Figure 6. Since only one emission peak was observed in the steady-state fluorescence, the major component (τ_2_ = 3.38 ns) was attributed to the emission from *keto* tautomer. It should be noted that the minor component (τ_1_ = 1.05 ns) might not be associated with the locally excited (LE) state of *enol* tautomer, as the fast rate of ESIPT (e.g., in less than 200 picoseconds) [16] makes it unlikely to have the lifetime in the nanosecond range. The assumption was consistent with the steady-state fluorescence, as no *enol* emission was observed (Figure 5).

It has been shown that pyridinium-containing styryl dyes typically exhibit a relatively short fluorescence lifetime. For example, 2-(4-(dimethylamino)styryl)-1-methylpyridinium iodide (DASPMI) in chloroform shows a three-exponential decay with τ_1_ = 34 and τ_2_ = 79 picoseconds, which are attributed to two different excited states, i.e., LE and ICT states [17]. In a polar solvent such as MeOH, DASPMI exhibits a three-exponential decay function, in which an additional component with a shorter lifetime (e.g., τ ≈ 1 ps) is assumed to be caused by solvation [18]. In a recent study by Spalletti and coworkers, [16] the pyridinium-containing dyes are further examined by ultrafast transient absorption, revealing a three-exponential decay in CH_2_Cl_2_ (Table 2), which includes LE, solvation and ICT processes. It should be noticed that the lifetimes of these pyridinium-containing styryl dyes are on the time scale of picoseconds. In sharp contrast to what is generally reported in the literature, the lifetime of **1** revealed only two components with significantly longer lifetimes (on the scale of nanoseconds) for LE and ICT, showing the significant impact of intramolecular hydrogen bonding on the lifetime parameter. 

Examination of compound **3** under the same condition also revealed similar fluorescence lifetime characteristics in CH_2_Cl_2_, exhibiting a two-exponential decay with τ_1_ = 2.04 ns (16%) and τ_2_ = 2.75 ns (84%) (Table 3). In order to shed some light on the observed fluorescence decay, model compound **5** was used in the study, which was synthesized as described in a previous report [8]. As shown in Scheme 1, the conjugation length of cyanine fragment in **3** can be approximated by compound **5**, as they exhibit nearly identical absorption λ_max_ (447 nm for **3** and 449 nm for **5** in CH_2_Cl_2_) [8]. Upon excitation with a 405 nm laser, however, the fluorescence lifetime measurement of **5** revealed a three-exponential decay (Table 3). With a chi-square value $\chi_{r}^{2}$ ≈1.0, the fitting gave two major components τ_1_ = 0.10 and τ_2_ = 0.24 ns (>98%) and a minor component τ_3_ ≈ 1.77 ns. The minor component (*τ_3_*) could be attributed to the ICT state since the intramolecular charge transfer (ICT) state typically had a significantly longer lifetime in styryl dyes (see Table 2). It should be noted that a dual fluorescence lifetime was expected from the excited state of a donor-acceptor stilbene [20]. One of the two components with shorter lifetimes (τ_1_ = 0.10 and τ_2_ = 0.24 ns) could be associated with the LE state.

**Scheme 3:**
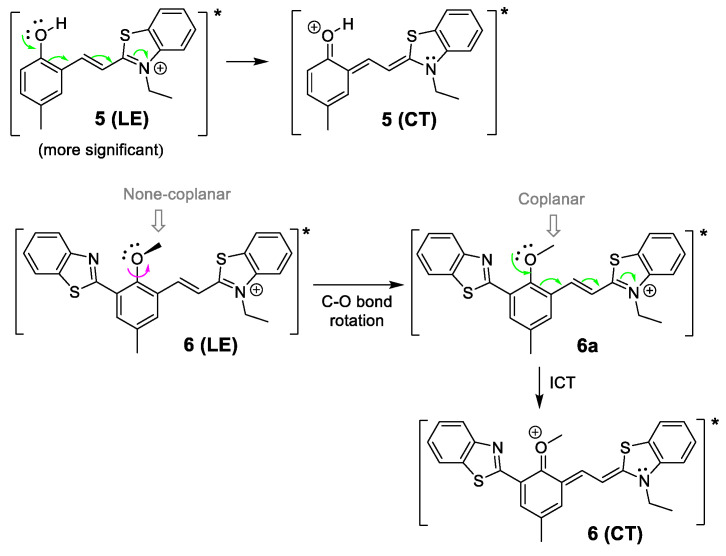
Schematic illustration of converting the locally excited (LE) state to charge transfer (CT) state by electron movement.

The fluorescence lifetime measurement of **6** also revealed a three-exponential decay in CH_2_Cl_2_, in agreement with **5**. Interestingly, multiexponential decay analysis of **6** revealed one minor component (τ_1_ ≈ 0.11 ns; fractional intensity ~5.3%) and two major components (τ_2_ ≈ 1.41 ns; τ_3_ ≈ 2.61 ns). The observed distribution from **6** was in contrast to that from **5**, whose distribution revealed two major components with shorter lifetimes (i.e., τ_1_ and τ_2_ less than 0.24 ns). Among the emissive species of **6**, the component with τ_3_ ≈ 2.67 ns was likely to be associated with the excited **6** (CT) that was the major emissive species. The assumption was consistent with the observed increase in its fractional intensity when the long path optical filter wavelength was increased from 550 nm to 650 nm, which allowed only the photons to be detected above 550 nm or 650 nm. The components with τ_1_ ≈ 0.1 ps and τ_2_ ≈ 1.45 ns could be attributed to the excited **6** (LE) and **6a**, respectively (Scheme 3). In summary, excited **3** exhibited a *two*-exponential decay, while its model compounds **5** and **6** showed a *three*-exponential decay. In other words, the results suggested that ESIPT functional group could play an important role in simplifying the fluorescence decay processes. 

**Scheme 4:**
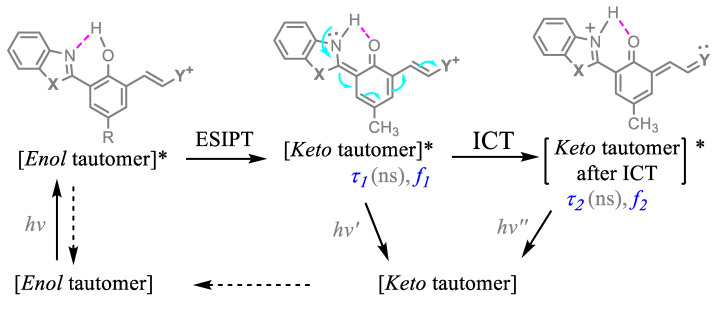
Schematic illustration of the connection between fluorescence lifetimes and ESIPT.

Previous studies have shown that HBT typically gives only one lifetime (on a picosecond time scale) that is associated with its *keto* tautomer [7]. Since the proton transfer in HBT has essentially no barrier and can occur at a very fast rate [5], it was unlikely to have the lifetime of the *enol* tautomer of **3** on a nanosecond scale. This pointed to the possibility that the singlet *keto* tautomer and its ICT species were potential lifetime components. The assumption was further supported by the observation of a multiexponential decay from **6** that cannot undergo ESIPT. On the basis of the above reasoning, the observed two lifetime components from **3** was likely to be associated with species that appeared after the ESIPT event. One possible species was the singlet *keto* tautomer, which was followed by another species after undergoing ICT, as shown in Scheme 4. The proposed decay involved the assumption that the deactivation of the excited states was basically determined by the styryl-cyanine fragment and ICT process. The assumption was consistent with the known literature examples of pyridinium-containing styryl dyes [19], whose lifetime components included LE and ICT (Table 2).

It should be noticed that all four compounds containing an ESIPT group (i.e., **1**–**4**) revealed a two-exponential decay (Table 3). For compounds **1**–**3**, their solution in CH_2_Cl_2_ gave a predominant excited component that involved the ICT process (with a fractional intensity as high as 94%). However, both components in the decay were present in significant fractional intensities (e.g., f_1_ *≈* 54% and f_2_ *≈* 46%) in CH_2_Cl_2_ for compound **4**. Observation of significant fractional contribution from both components indicated that none of them could be associated with the *enol* tautomer since the steady-state fluorescence of **4** observed only *keto* emission [10]. The large variation in the fractional contribution could be related to the extent of ICT interaction, which deserves further investigation.

## 3. Materials and Methods

All starting materials and the essential solvents were purchased from Sigma-Aldrich, TGI, Ark Pharma, Fischer Scientific, Alfa-Asaer and Across Organics and directly used without further purification. Starting materials 2-(Benzo[d]thiazol-2-yl)-4-methylphenol (**8**) and 2-(benzo[d]oxazol-2-yl)-4-methylphenol (**10**) were synthesized by using literature procedures. All deuterated solvents were purchased from Cambridge Isotopes and used as received. All NMR data were recorded on Varian 300 and 500 MHz instruments with all spectra referenced to deuterated solvents. HRMS data were acquired on an ESI-TOF MS system (Waters, Milford, MA). UV−vis studies were carried out in Hewlett-Packard-8453 diode array-based spectrophotometer at 25 °C. Fluorescence spectral analysis was conducted by using a HORIBA Fluoromax-4 spectrofluorometer. 

Fluorescence lifetime was measured by using a *time-correlated single-photon counting* (TCSPC) method, on a Horiba DeltaPro lifetime system, which is capable of measuring a lifetime range of 30 ps-1 s. The instrument is equipped with a picosecond photon detection module comprising a fast, cooled, photomultiplier with 230–850nm response. All measurements were performed by exciting the sample solutions with a Horiba DeltaDiode^TM^ DD-405 Laser (peak wavelength at 405 nm +/−10 nm).

## 4. Conclusions

In conclusion, the fluorescence lifetimes of compounds **1**–**4** have been determined by using *time-correlated single-photon counting* (TCSPC) method (Figure 6; ESI Tables S1–S4, Figures S29–S39). A polar aprotic solvent, such as CH_2_Cl_2_, was used in the study, which provides good solubility while avoiding the phenolic proton dissociation that could lead to a new component to complicate the study. The experimental study revealed two lifetime components in the range of 1.0–3.6 nanoseconds (ns), depending on the structure of terminal cyanine segments. The identified lifetime components were mainly associated with the excited *keto* tautomers, as only keto emission was observed in the steady-state fluorescence spectra. For each compound, the lifetime difference (τ_2_ − τ_1_) in the two identified components also exhibited significant differences, ranging from 0.7 ns for **3** to 2.33 ns for **1**, which is in agreement with the large structural difference between the probes.

The study was further carried out by comparison of **3** with its structural similar model styryl compounds **5**–**6**, in which the *intramolecular proton transfer* is no longer possible. Evaluation of their fluorescence decay revealed *three* lifetime constants for **5**–**6** (ESI Tables S5–S8, Figures S40–S51), in contrast to *two* for **3**. For example, the observed three lifetimes from **6** were τ_1_ ≈ 0.1, τ_2_ ≈ 1.4, and τ_3_ ≈ 2.6 ns. Our study suggested that the component with the longest lifetime (τ_3_ ≈ 2.6 ns) could be attributed to the ICT, while the component with the shortest lifetime to the locally excited **6** (LE) (Scheme 3). A molecular modeling study further confirmed that the methoxy group in the ground state of **6** was not coplanar with the attached aromatic ring (ESI Figure S52), which could be responsible for the additional lifetime component (τ_1_ ≈ 0.1). Thus, intramolecular hydrogen bonding played an essential role in maintaining molecular *co*-planarity, which is desirable in the excited state (for ICT) and responsible for a simplified decay pathway.

The experimental finding showed that the excited state of ESIPT compounds **1**–**4** exhibited a two-exponential decay in a polar aprotic solvent, in contrast to the mono-exponential decay typically observed from HBT. The multiexponential decay of **1**–**4** was consistent with the known cyanine-containing styryl dyes (e.g., DASPMI) and model compounds **5**–**6**. The results showed the large impact of the cyanine segment that contributed to the enhanced ICT interaction in the excited states. The ICT interaction in these ESIPT compounds had a significant impact on the fluorescence lifetime characteristics, including lifetime parameters (τ_i_) and fractional intensities (f_i_). These findings will provide useful data to guide the further development of this class of materials.

## Figures and Tables

**Table 1:** Photophysical properties of compounds in different solvents.

Compound	Methylene Chloride			Acetonitrile			Methanol		
	λ_abs_/nm	λ_em_/nm	Φ_fl_	λ_abs_/nm	λ_em_/nm	Φ_fl_	λ_abs_/nm	λ_em_/nm	Φ_fl_
**1**	421	686	0.34	397	690	0.19	401	695	0.18
**2**	413	678	0.27	391	683	0.28	394	693	0.14
**3**	447	683	0.32	423	704	0.21	423	707	0.15
**4**	436	675	0.28	417	700	0.19	416	692	0.15
**5**	447	580	0.009	416	568	0.008	425	576	0.007
**6**	399	587/660	0.0021	379	575/696	0.0020	382	572/690	0.0015

**Table 2:** Fluorescence lifetime of two pyridinium-containing styryl dyes in CH_2_Cl_2_ (from reference [19], Copyright © 2022, American Chemical Society).

Pyridinium-Based Stilbenes ^a^	Lifetime (ps)	Transient
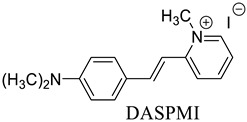	τ_1_ = 0.70	Solv
	τ_2_ = 52	^1^LE ^b^
	τ_3_ = 134	^1^ICT
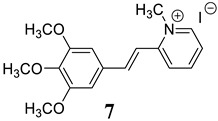	τ_1_ = 0.68	Solv
	τ_2_ = 10	^1^LE
	τ_3_ = 172	^1^ICT

^a^ Chemical structure of two pyridinium-based donor–acceptor stilbenes: *o*-DASPMI [17] and I-1-methyl-2-(3,4,5-trimethoxystyryl)pyridine-1-ium **7**. [19] ^b 1^LE and ^1^ICT denote *singlet* LE and ICT excited states.

**Table 3:** Fluorescence lifetime data of ESIPT compounds in CH_2_Cl_2_.

Compound	Lifetime (ns)			Normalized Pre-Exponential			Fractional Intensities			$\bar{\tau }\;$ (ns)	χ^2^
	τ_1_	τ_2_	τ_3_	α_1_	α_2_	α_3_	f_1_	f_2_	f_3_		
**1**	1.05	3.38		0.17	0.83		5.84%	94.16%		2.99	1.02
**2**	1.27	3.61		0.14	0.86		5.52%	94.48%		3.27	1.03
**3**	2.04	2.75		0.20	0.80		15.84%	84.16%		2.60	1.14
**4**	1.70	3.07		0.68	0.32		53.97%	46.03%		2.14	1.02
**5**	0.10	0.24	1.77	0.53	0.47	0.00	32.04%	66.62%	1.33%	0.17	1.00
**6** *	0.11	1.41	2.61	0.45	0.43	0.12	5.30%	63.24%	31.47%	0.96	1.12
**6** **	0.10	1.35	2.67	0.27	0.47	0.26	1.99%	46.74%	51.27%	1.35	1.17

* Data obtained with a 550 nm long path filter, by using the same conditions as for **1**–**5**; ** data obtained with a 650 nm long path filter, and $\bar{\tau }$ represents the average lifetime, $\bar{\tau }=\alpha_{1}\tau_{1}+\alpha_{2}\tau_{2}+\alpha_{3}\tau_{3}$.

## Data Availability

Not applicable.

## Supplementary Materials

The following supporting information can be downloaded at: https://www.mdpi.com/article/10.3390/molecules28010125/s1. Figure S1. ^1^H-NMR spectra of compound **9** in CDCl_3_. Figure S2. 1H-NMR spectra of compound **11** in CDCl_3_. Figure S3. 1H-NMR spectra of compound **1** in DMSO-d6. Figure S4. 1H-NMR spectra of compound **2** in DMSO-d_6_. Figure S5. 1H-NMR spectra of compound **3** in DMSO-d_6_. Figure S6. 1H-NMR spectra of compound **4** in DMSO-d_6_. Figure S7. 1H-NMR spectra of compound **5** in DMSO-d_6_. Figure S8. 1H-NMR spectra of compound **6** in DMSO-d_6_. Figure S9. ^13^C-NMR spectra of compound **6** in DMSO-d_6_. Figure S10. ESI-MS spectra of compound **6**. Figure S11. UV-vis absorption (solid line) and emission spectra (broken line) of compound **1** in DCM (10 μM). The excitation wavelength was 430 nm. Figure S12. UV-vis absorption (solid line) and emission spectra (broken line) of compound **1** in MeCN (10 μM). The excitation wavelength was 400 nm. Figure S13. UV-vis absorption (solid line) and emission spectra (broken line) of compound **1** in MeOH (10 μM). The excitation wavelength was 400 nm. Figure S14. UV-vis absorption (solid line) and emission spectra (broken line) of compound **2** in DCM (10 μM). The excitation wavelength was 410 nm. Figure S15. UV-vis absorption (solid line) and emission spectra (broken line) of compound **2** in MeCN (10 μM). The excitation wavelength was 390 nm. Figure S16. UV-vis absorption (solid line) and emission spectra (broken line) of compound **2** in MeOH (10 μM). The excitation wavelength was 400 nm. Figure S17. UV-vis absorption (solid line) and emission spectra (broken line) of compound **3** in DCM (10 μM). The excitation wavelength was 450 nm. Figure S18. UV-vis absorption (solid line) and emission spectra (broken line) of compound 3 in MeCN (10 μM). The excitation wavelength was 430 nm. Figure S19. UV-vis absorption (solid line) and emission spectra (broken line) of compound **3** in MeOH (10 μM). The excitation wavelength was 430 nm. Figure S20. UV-vis absorption (solid line) and emission spectra (broken line) of compound **4** in DCM (10 μM). The excitation wavelength was 440 nm. Figure S21. UV-vis absorption (solid line) and emission spectra (broken line) of compound **4** in MeCN (10 μM). The excitation wavelength was 420 nm. Figure S22. UV-vis absorption (solid line) and emission spectra (broken line) of compound **4** in MeOH (10 μM). The excitation wavelength was 420 nm. Figure S23. UV-vis absorption (solid line) and emission spectra (broken line) of compound **5** in DCM (10 μM). The excitation wavelength was 450 nm. Figure S24. UV-vis absorption (solid line) and emission spectra (broken line) of compound **5** in MeCN (10 μM). The excitation wavelength was 410 nm. Figure S25. UV-vis absorption (solid line) and emission spectra (broken line) of compound **5** in MeOH (10 μM). The excitation wavelength was 430 nm. Figure S26. UV-vis absorption (solid line) and emission spectra (broken line) of compound **6** in MeOH (10 μM). The excitation wavelength was 400 nm. Figure S27. UV-vis absorption (solid line) and emission spectra (broken line) of compound **6** in MeCN (10 μM). The excitation wavelength was 385 nm. Figure S28. UV-vis absorption (solid line) and emission spectra (broken line) of compound **6** in MeOH (10 μM). The excitation wavelength was 385 nm. Table S1. Fluorescence lifetime data of compound **1**. Table S2. Fluorescence lifetime data of compound **2**. Table S3. Fluorescence lifetime data of compound **3**. Table S4. Fluorescence lifetime data of compound **4**. Table S5. Fluorescence lifetime data of compound **5** (two-exponential). Table S6. Fluorescence lifetime data of compound **5** (three-exponential). Table S7. Fluorescence lifetime data of compound **6** (550 nm long path filter). Table S8. Fluorescence lifetime data of compound **6** (650 nm long path filter). Figure S29. The fluorescence lifetime of IRF (purple square) and decay for compound **1** in MeCN (green circle) and fitted curve of two exponential functions. The residuals are reported in the lower panel. With DD-405L (λ_em_ = 406 nm) as light source and 650 nm long path filter. Figure S30. The fluorescence lifetime of IRF (purple square) and decay for compound **1** in MeOH (green circle) and fitted curve of two exponential functions. The residuals are reported in the lower panel. With DD-405L (λ_em_ = 406 nm) as light source and 650 nm long path filter. Figure S31. The fluorescence lifetime of IRF (purple square) and decay for compound **2** in DCM (green circle) and fitted curve of two exponential functions. The residuals are reported in the lower panel. With DD-405L (λ_em_ = 406 nm) as light source and 650 nm long path filter. Figure S32. The fluorescence lifetime of IRF (purple square) and decay for compound **2** in MeCN (green circle) and fitted curve of two exponential functions. The residuals are reported in the lower panel. With DD-405L (λ_em_ = 406 nm) as light source and 650 nm long path filter. Figure S33. The fluorescence lifetime of IRF (purple square) and decay for compound **2** in MeOH (green circle) and fitted curve of two exponential functions. The residuals are reported in the lower panel. With DD-405L (λ_em_ = 406 nm) as light source and 650 nm long path filter. Figure S34. The fluorescence lifetime of IRF (purple square) and decay for compound **3** in DCM (green circle) and fitted curve of two exponential functions. The residuals are reported in the lower panel. With DD-405L (λ_em_ = 406 nm) as light source and 650 nm long path filter. Figure S35. The fluorescence lifetime of IRF (purple square) and decay for compound **3** in MeCN (green circle) and fitted curve of two exponential functions. The residuals are reported in the lower panel. With DD-405L (λ_em_ = 406 nm) as light source and 650 nm long path filter. Figure S36. The fluorescence lifetime of IRF (purple square) and decay for compound **3** in MeOH (green circle) and fitted curve of two exponential functions. The residuals are reported in the lower panel. With DD-405L (λ_em_ = 406 nm) as light source and 650 nm long path filter. Figure S37. The fluorescence lifetime of IRF (purple square) and decay for compound **4** in DCM (green circle) and fitted curve of two exponential functions. The residuals are reported in the lower panel. With DD-405L (λ_em_ = 406 nm) as light source and 650 nm long path filter. Figure S38. The fluorescence lifetime of IRF (purple square) and decay for compound **4** in MeCN (green circle) and fitted curve of two exponential functions. The residuals are reported in the lower panel. With DD-405L (λ_em_ = 406 nm) as light source and 650 nm long path filter. Figure S39. The fluorescence lifetime of IRF (purple square) and decay for compound **4** in MeOH (green circle) and fitted curve of two exponential functions. The residuals are reported in the lower panel. With DD-405L (λ_em_ = 406 nm) as light source and 650 nm long path filter. Figure S40. The fluorescence lifetime of IRF (purple square) and decay for compound **5** in DCM (green circle) and fitted curve of two exponential functions. The residuals are reported in the lower panel. With DD-405L (λ_em_ = 406 nm) as light source and 550 nm long path filter. Figure S41. The fluorescence lifetime of IRF (purple square) and decay for compound **5** in MeCN (green circle) and fitted curve of two exponential functions. The residuals are reported in the lower panel. With DD-405L (λ_em_ = 406 nm) as light source and 550 nm long path filter. Figure S42. The fluorescence lifetime of IRF (purple square) and decay for compound **5** in MeOH (green circle) and fitted curve of two exponential functions. The residuals are reported in the lower panel. With DD-405L (λ_em_ = 406 nm) as light source and 550 nm long path filter. Figure S43. The fluorescence lifetime of IRF (purple square) and decay for compound **5** in DCM (green circle) and fitted curve of three exponential functions. The residuals are reported in the lower panel. With DD-405L (λ_em_ = 406 nm) as light source and 550 nm long path filter. Figure S44. The fluorescence lifetime of IRF (purple square) and decay for compound **5** in MeCN (green circle) and fitted curve of three exponential functions. The residuals are reported in the lower panel. With DD-405L (λ_em_ = 406 nm) as light source and 550 nm long path filter. Figure S45. The fluorescence lifetime of IRF (purple square) and decay for compound **5** in MeOH (green circle) and fitted curve of three exponential functions. The residuals are reported in the lower panel. With DD-405L (λ_em_ = 406 nm) as light source and 550 nm long path filter. Figure S46. The fluorescence lifetime of IRF (purple square) and decay for compound **6** in DCM (green circle) and fitted curve of three exponential functions. The residuals are reported in the lower panel. With DD-405L (λ_em_ = 406 nm) as light source and 550 nm long path filter. Figure S47. The fluorescence lifetime of IRF (purple square) and decay for compound **6** in MeCN (green circle) and fitted curve of three exponential functions. The residuals are reported in the lower panel. With DD-405L (λ_em_ = 406 nm) as light source and 550 nm long path filter. Figure S48. The fluorescence lifetime of IRF (purple square) and decay for compound **6** in MeOH (green circle) and fitted curve of three exponential functions. The residuals are reported in the lower panel. With DD-405L (λ_em_ = 406 nm) as light source and 550 nm long path filter. Figure S49. The fluorescence lifetime of IRF (purple square) and decay for compound **6** in DCM (green circle) and fitted curve of three exponential functions. The residuals are reported in the lower panel. With DD-405L (λ_em_ = 406 nm) as light source and 650 nm long path filter. Figure S50. The fluorescence lifetime of IRF (purple square) and decay for compound **6** in MeCN (green circle) and fitted curve of three exponential functions. The residuals are reported in the lower panel. With DD-405L (λ_em_ = 406 nm) as light source and 650 nm long path filter. Figure S51. The fluorescence lifetime of IRF (purple square) and decay for compound **6** in MeOH (green circle) and fitted curve of three exponential functions. The residuals are reported in the lower panel. With DD-405L (λ_em_ = 406 nm) as light source and 650 nm long path filter. Figure S52. The simulated molecular geometry of compound **6** on the ground state via DFT method (a) and on the excited state via TD-DFT method (b) at B3LYP/6-31G (d, p) level. Figure S53. UV-vis absorption (solid line) and emission spectra (broken line) of compound **6** (10 μM) in Ethylene Glycol (MEG). The excitation wavelength was 385 nm. Figure S54. The fluorescence lifetime of IRF (purple square) and decay for compound **6** in MEG (green circle) and fitted curve of three exponential functions. The residuals are reported in the lower panel. With DD-405L (λ_em_ = 406 nm) as light source and 550 nm long path filter. Figure S55. The fluorescence lifetime of IRF (purple square) and decay for compound **6** in MEG (green circle) and fitted curve of three exponential functions. The residuals are reported in the lower panel. With DD-405L (λ_em_ = 406 nm) as light source and 650 nm long path filter.

## Sample Availability

Samples of the compounds **1**–**6** are available from the authors.
