# Supplementary material for: Fluorescence Lifetimes of NIR-Emitting Molecules with Excited-State Intramolecular Proton Transfer
Source: Molecules. 2022 Dec 23;28(1):125. doi: 10.3390/molecules28010125 (PMC9822172; doi:10.3390/molecules28010125)
Supplement: Supplementary file 1 [file molecules-28-00125-s001.zip › molecules-2114515-supplementary.pdf]

Fluorescence Lifetimes of NIR-Emitting Molecules with Excited-State Intramolecular Proton Transfer

Yonghao Li, Dipendra Dahal and Yi Pang *

Department of Chemistry, University of Akron, Akron, OH 44325, USA

*Correspondence: yp5@uakron.edu

Synthesis of 3-(benzo[d]thiazol-2-yl)-2-hydroxy-5-methyl- benzaldehyde (**9**).

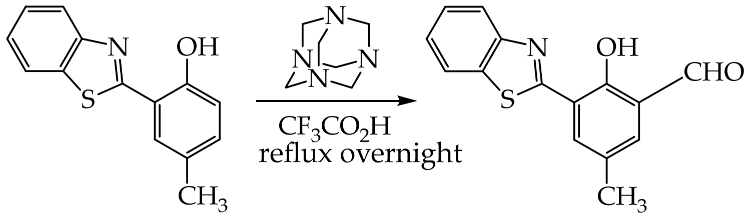

8 9

2-(Benzo[d]thiazol-2-yl)-4-methylphenol (**8**), hexamethylenetetramine and trifluoroacetic acid were added to a round bottom flask and refluxed overnight. After the mixture was cooled down, neutralized the acid with saturated KOH solution. The precipitate was collected by filtration and washed with water several times. After drying under vacuum, compound **9** was obtained.

^{1}H-NMR (300 MHz, $CDCl_3$): δ 13.04 (s,1H), δ 10.50 (s, 1H), δ 8.04 (d, 1H), δ 7.93 (t, 2H), δ 7.71 (s, 1H), δ 7.54 (t, 1H), δ 7.45 (t, 1H), δ 2.41 (s, 3H).

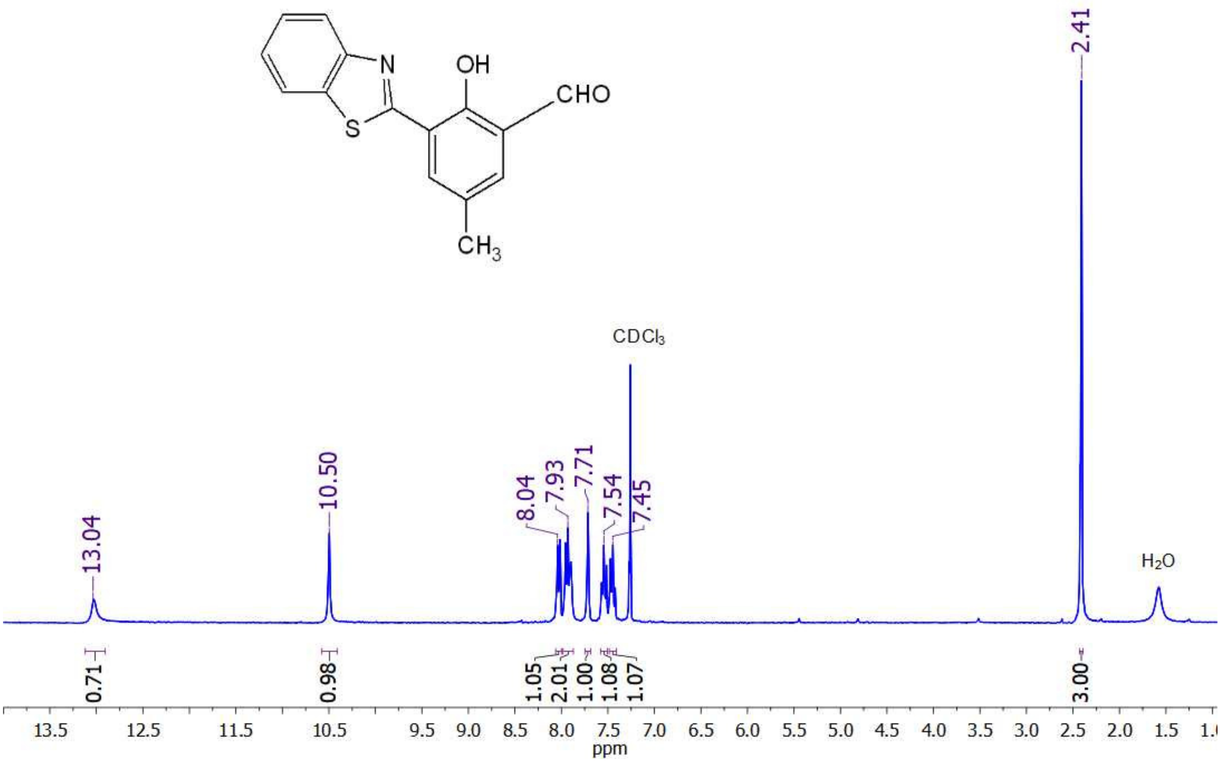

Figure S1. ^{1}H-NMR spectra of compound **9** in $CDCl_3$

Synthesis of 3-(benzo[d]oxazol-2-yl)-2-hydroxy-5-methylbenzaldehyde (**11**).

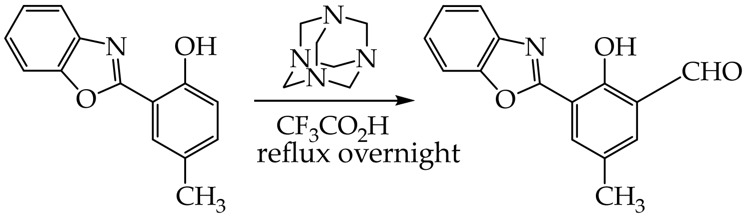

10 11

Compound **10**, hexamethylenetetramine and trifluoroacetic acid were added to a round bottom flask and refluxed overnight. After the mixture was cooled down, neutralized the acid with saturated KOH solution. The precipitate was collected by filtration and washed with water several times. After drying under vacuum, compound **11** was obtained.

^{1}H-NMR (300 MHz, $CDCl_3$): δ 10.60 (s, 1H), δ 8.11 (s, 1H), δ 7.81 (m, 2H), δ 7.64 (m, 1H), δ 7.44 (m, 2H), δ 2.42 (s, 3H).

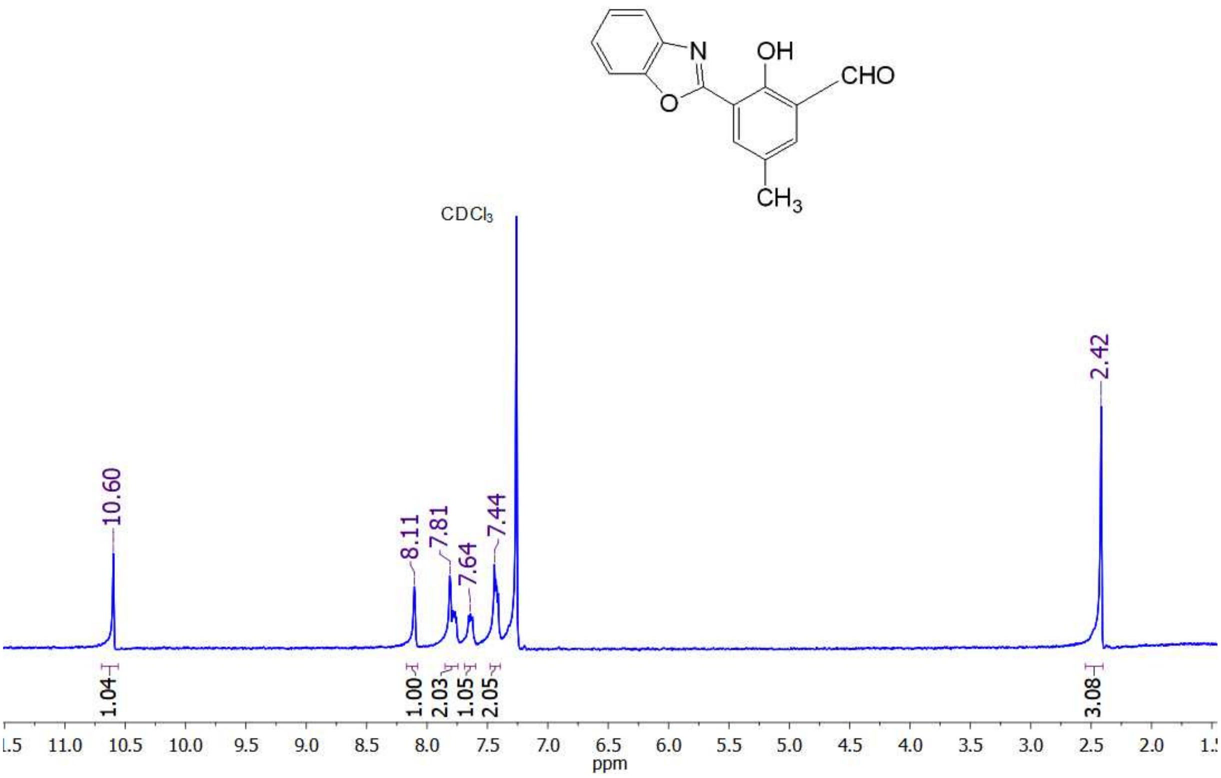

Figure S2. ^{1}H-NMR spectra of compound **11** in $CDCl_3$

Synthesis of (E)-4-(3-(benzo[d]thiazol-2-yl)-2-hydroxy-5methylstyryl)-1-methylpyridin-1-ium (**1**)

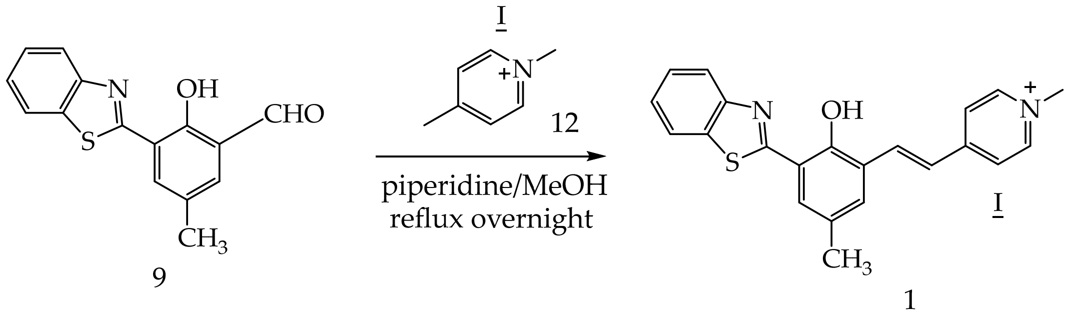

1,4-Dimethylpyridinium iodide (**12**) and piperidine were dissolved into MeOH, the mixture was stirred for 20 minutes and 3-(benzo[d]thiazol-2-yl)-2-hydroxy-5-methyl- benzaldehyde (**9**) was added and the final mixture was stirred and refluxed overnight. The solvent was evaporated by rotary evaporation. The resulting solid was washed with ethyl acetate and filtered out. Then it was washed with water and completely dried under a high vacuum to yield product **1**.

1H NMR (500 MHz, DMSO-d_6) δ 8.78 (d, 2H), δ 8.17 (d, 3H), δ 8.09 (d, 1H), 8.05 (d,1H), 7.9 (s, 1H), 7.84 (s,1H), 7.68 (t, 2H), 7.57 (t, 1H), 7.49 (t,1H), 4.24 (s, 3H), 2.37 (s, 3H).

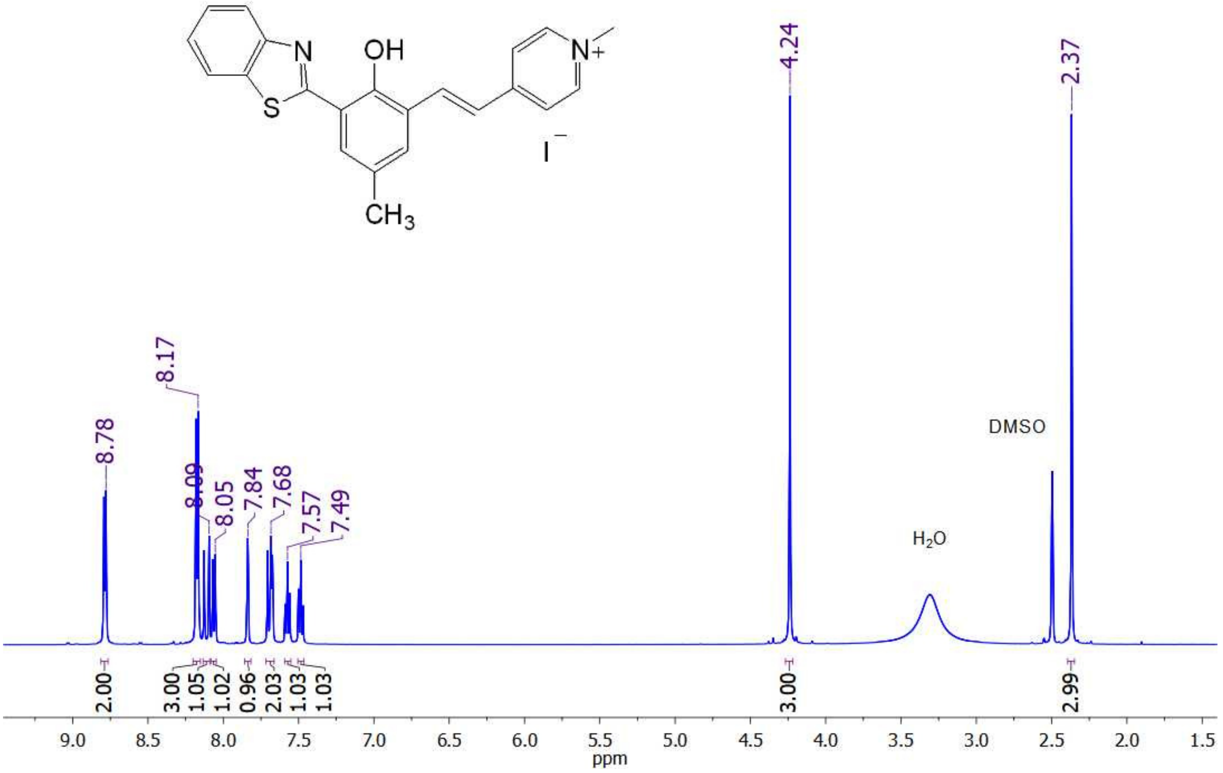

Figure S3. ^{1}H-NMR spectra of compound **1** in DMSO-d$_6$.

Synthesis of €-4-(3-(benzo[d]oxazol-2-yl)-2-hydroxy-5-methylstyryl)-1-methylpyridin-1-ium iodide (**2**)

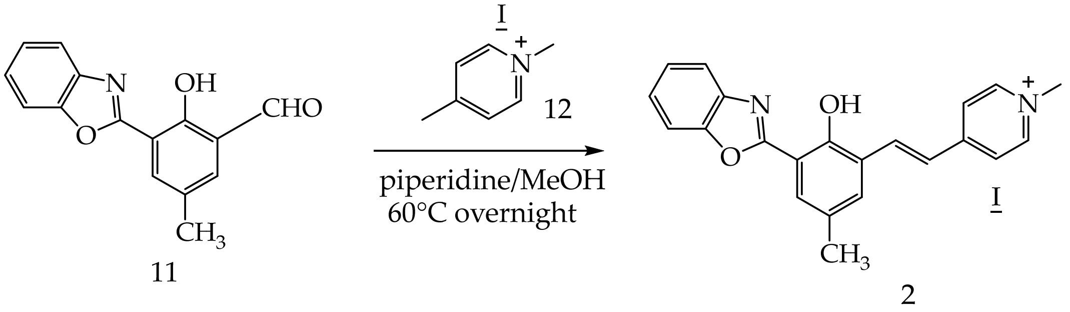

1,4-Dimethylpyridinium iodide (**12**) and piperidine were dissolved into MeOH, the mixture was stirred for 20 minutes and 3-(benzo[d]oxazol-2-yl)-2-hydroxy-5-methylbenzaldehyde (**11**) was added and the final mixture was stirred at 60°C overnight. After evaporating the solvent by a rotary evaporator, the solid residue was collected by filtration, washed with ethyl acetate, and dried under vacuum to yield product **2**.

^{1}H-NMR (500 MHz, DMSO-d$_6$) δ= 12.04 (s, 1H), 8.82 (d, 2H), 8.22 (d, 2H), 8.07 (d, 1H), 7.92 (s, 1H), 7.85 (t, 2H), 7.79 (s, 1H), 7.69 (d, 1H), 7.49 (m, 2H), 4.26 (s, 3H), 2.38 (s, 3H).

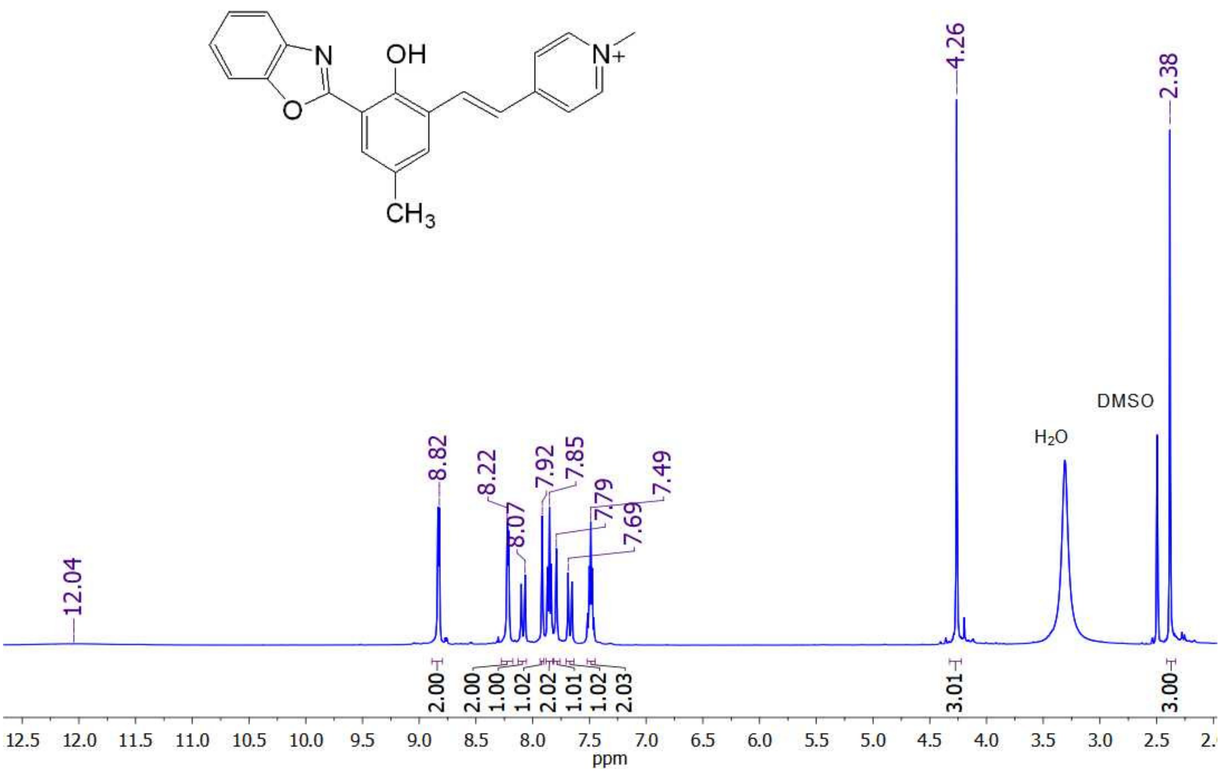

Figure S4. ^{1}H-NMR spectra of compound **2** in DMSO-d_6.

Synthesis of compound 2-[3-(benzo[d]thiazol-2-yl)-2-hydroxy-5-methylstyr-yl]-3- ethylbenzo[d]thiazol-3-ium iodide (**3**).

3-(benzo[d]thiazol-2-yl)-2-hydroxy-5-methyl- benzaldehyde (**9**), 3-Ethyl-2-methylbenzothiazolium iodide (**13**) and pyridine were added in methanol and refluxed overnight. After evaporating the solvent by a rotary evaporator, the solid residue was collected by filtration, washed with ethyl acetate, and dried under vacuum to give brown solid product **3**.

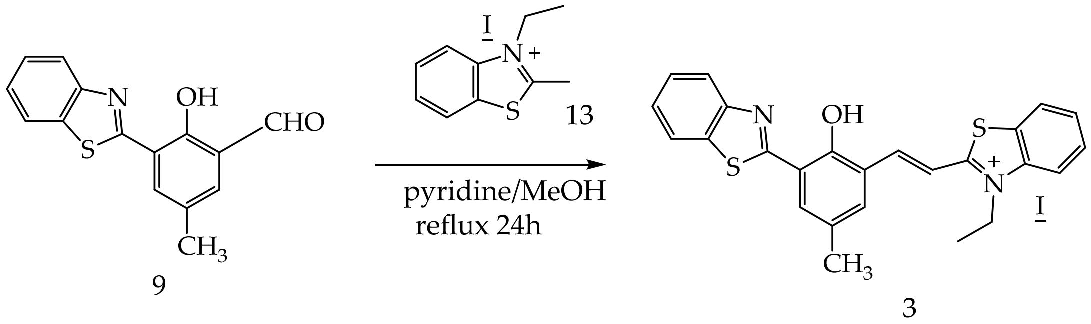

^{1}H-NMR (500 MHz, DMSO-d_6): δ 13.37 (s, 1H), δ 8.44 (d, 1H), δ 8.35 (d, 1H), δ 8.32 (d, 1H), δ 8.23(d, 1H), δ 8.15 (s, 1H), δ 8.13 (t, 2H), δ 7.96 (s, 1H), δ 7.89 (t, 1H), δ 7.81 (t, 1H), δ 7.62 (t, 1H), δ 7.54 (t, 1H), δ 4.98 (q, 2H), δ 2.45 (s, 3H), δ 1.52 (t, 3H).

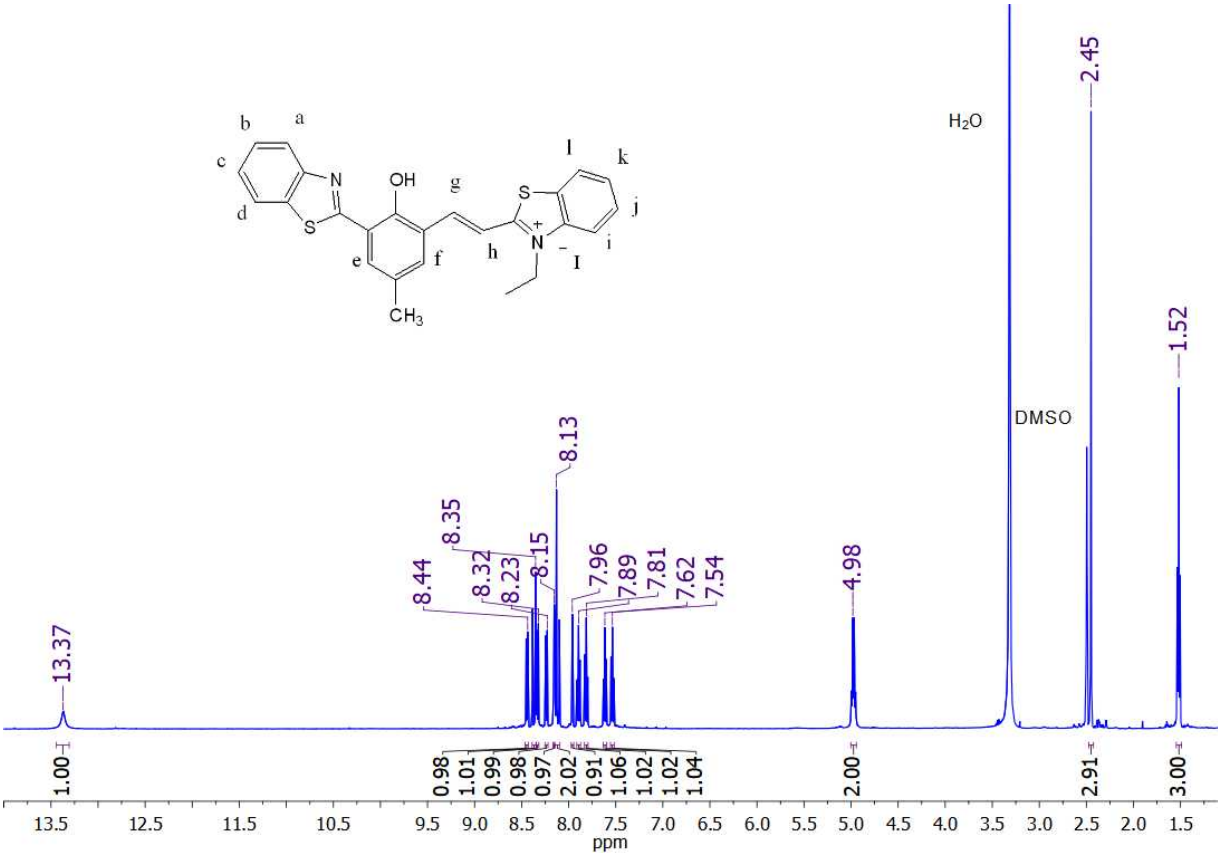

Figure S5. ^{1}H-NMR spectra of compound **3** in DMSO-d$_6$

Synthesis of (E)-4-(3-(benzo[d]thiazol-2-yl)-2-hydroxy-5methylstyryl)-1-methylpyridin-1-ium (**4**)

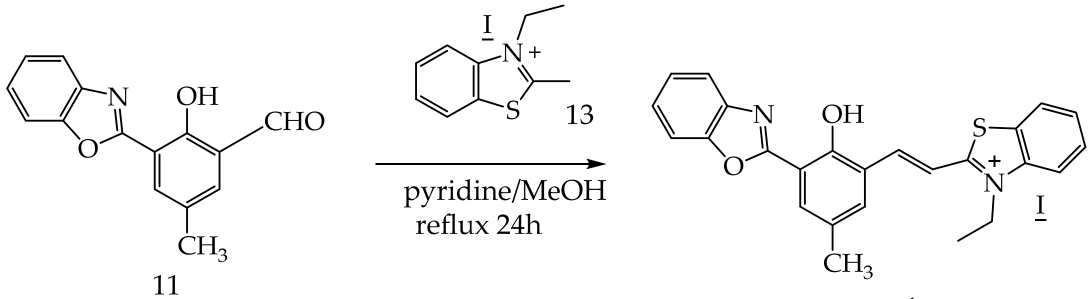

3-Ethyl-2-methylbenzothiazolium iodide (**13**) and pyridine were dissolved into MeOH, the mixture was stirred for 20 minutes and 3-(benzo[d]oxazol-2-yl)-2-hydroxy-5-methylbenzaldehyde (**11**) was added and refluxed overnight. After evaporating the solvent by a rotary evaporator, the solid residue was collected by filtration, washed with ethyl acetate, and dried under vacuum to yield product **4**.

^{1}H-NMR (500 MHz, DMSO-d$_6$): δ 12.30 (s,1H), δ 8.43 (d,1H), δ 8.33 (t,2H), δ 8.19 (s,1H), δ 8.15 (d,1H), δ 8.06 (s,1H), δ 7.88 (m,3H), δ 7.81 (t,1H), δ 7.51(m,2H), δ 4.96 (q,2H), δ 2.45(s, 3H), δ 1.52(t,3H).

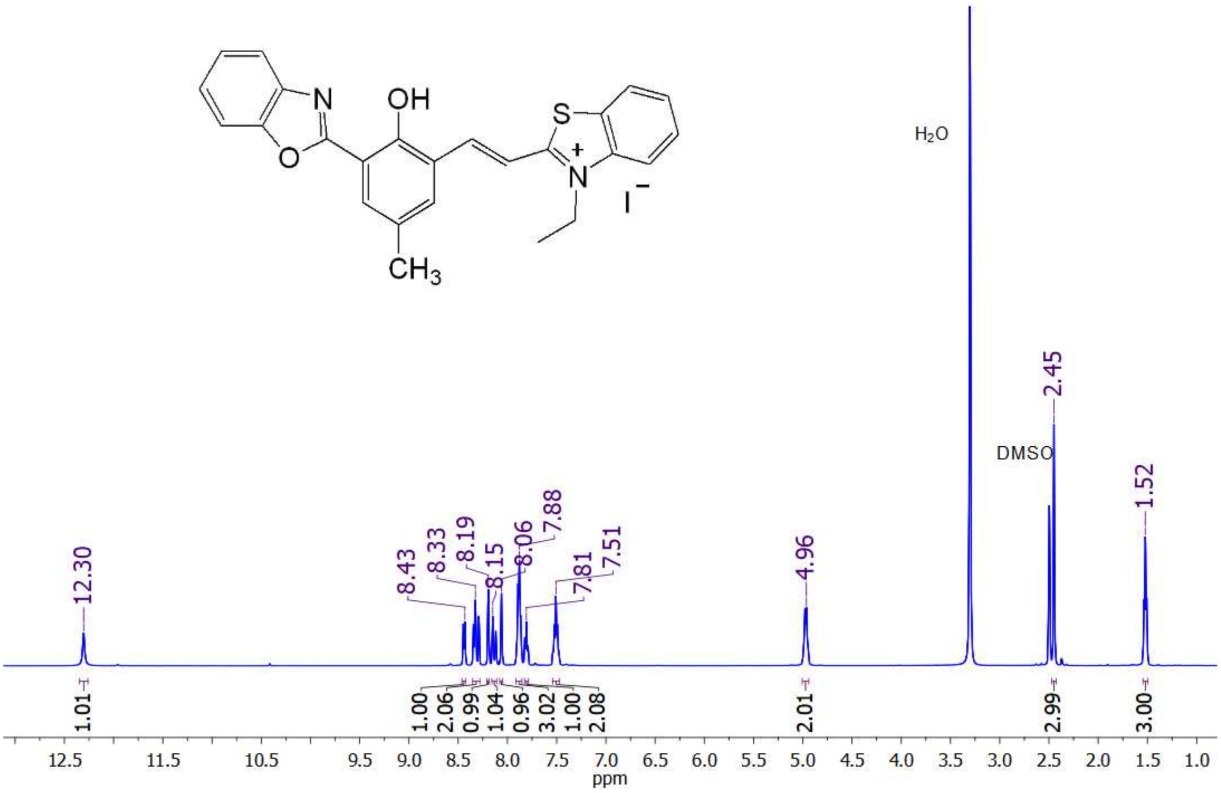

Figure S6. ^{1}H-NMR spectra of compound **4** in DMSO-d$_6$

Synthesis of 3-ethyl-2-(hydroxy-5-methylstyryl) benzo[d]thiazol-3-ium iodide (**5**).

2-hydroxy-5-methylbenzaldehyde (**14**) and 3-Ethyl-2-methylbenzothiazolium iodide (**13**) as well as pyridine were added to the methanol and refluxed overnight. After evaporating solvent by a rotary evaporator, product **5** was washed with ethyl acetate, filtered out and dried under vacuum.

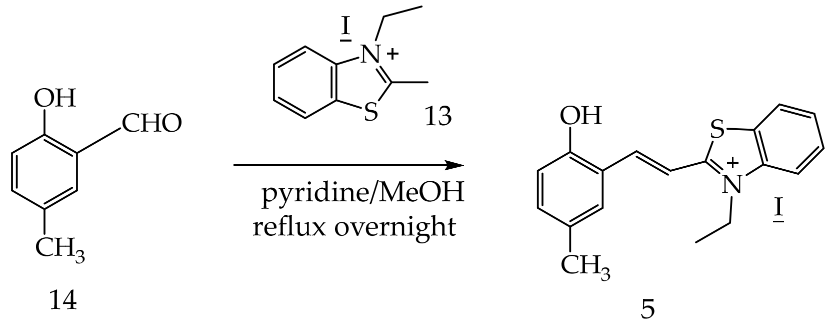

^{1}H-NMR (300 MHz, DMSO-d$_6$): δ 10.63 (s, 1H), δ 8.36 (d, 1H), δ 8.29 (s, 1H), δ 8.24 (d, 1H), 7.85 (m, 4H), 7.22 (d, 1H), δ 6.92 (d, 1H), δ 4.92 (q, 2H), δ 2.28 (s, 3H), δ 1.47 (t, 3H).

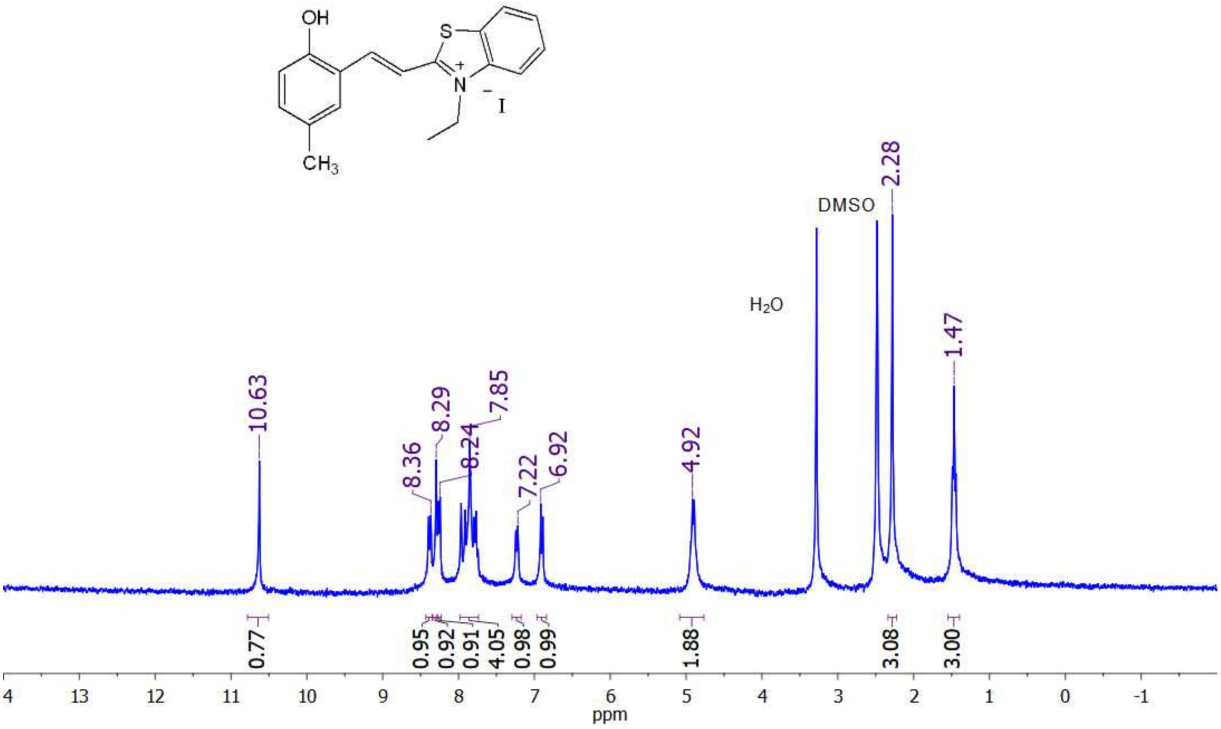

Figure S7. ^{1}H-NMR spectra of compound **5** in DMSO-d6

Synthesis of (E)-2-(3-(benzo[d]thiazol-2-yl)-2-methoxy-5-methylstyryl)-3-ethylbenzo[d]thiazol-3-ium iodide (**6**)

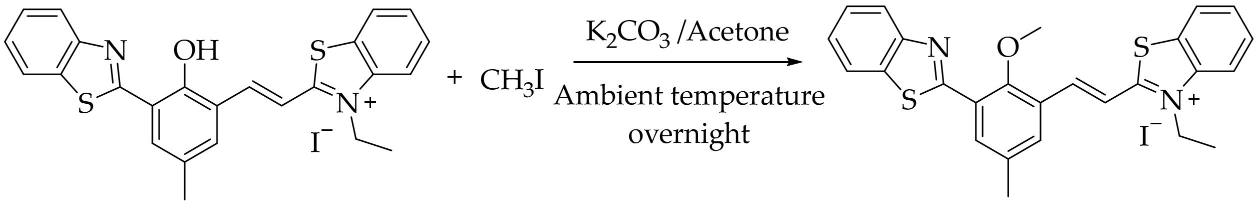

3

6

In a 50 mL RB flask equipped with a magnetic stir bar, 30 mL acetone was taken under argon protection. 300 mg compound **3** was added to the acetone, and 186.2 mg K_2CO_3 (2.0 eq. 1.348 mmol) was added under argon protection. 153 mg methyl iodide (2.0 eq, 1.078 mmol) was added using the syringe, and the mixture was stirred overnight at ambient temperature. After completion of the reaction, the mixture was diluted with 30 mL acetone and filtered off to remove the solid K_2CO_3, and the residue was washed with 20 mL acetone. Dry the filtrate with a rotary evaporator. The solid residue was slurry in 30 mL ethyl acetate and stirred for 1 hour. The slurry was filtered off using a vacuum filter, and the residue was washed with 30 mL of ethyl acetate. The solid was vacuum dried and a dark brown/red colored solid was obtained.

^{1}H-NMR (500 MHz, DMSO-d$_6$): δ 8.48 (d, 1H), δ 8.39 (d, 2H), δ 8.30 (s, 1H), δ 8.20 (q, 3H), δ 8.10 (d, 1H), δ 7.92 (t, 1H), δ 7.84 (t, 1H), δ 7.58 (t, 1H), δ 7.50 (t, 1H), δ 5.04 (q, 2H), δ 3.93 (s, 3H), δ 2.52 (s, 3H), δ 1.53 (t, 3H).

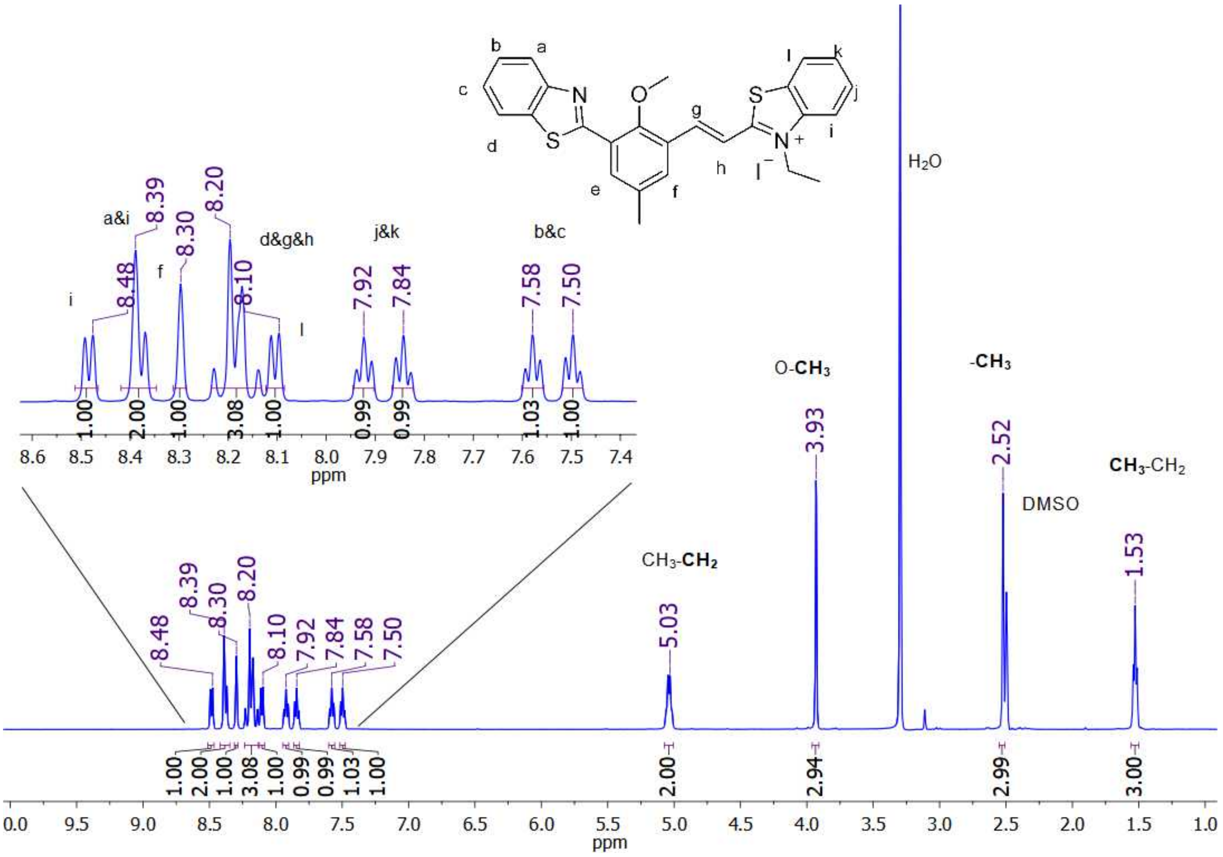

Figure S8. ^{1}H-NMR spectra of compound **6** in DMSO-d$_6$

^{13}C-NMR (126 MHz, DMSO-d$_6$) δ 171.99, 161.64, 156.12, 152.12, 141.89, 141.50, 135.97, 135.46, 133.59, 132.41, 130.24, 129.17, 129.03, 128.47, 127.06, 127.01, 126.06, 125.05, 123.26, 122.58, 117.40, 115.84, 64.63, 45.43, 20.87, 14.90.

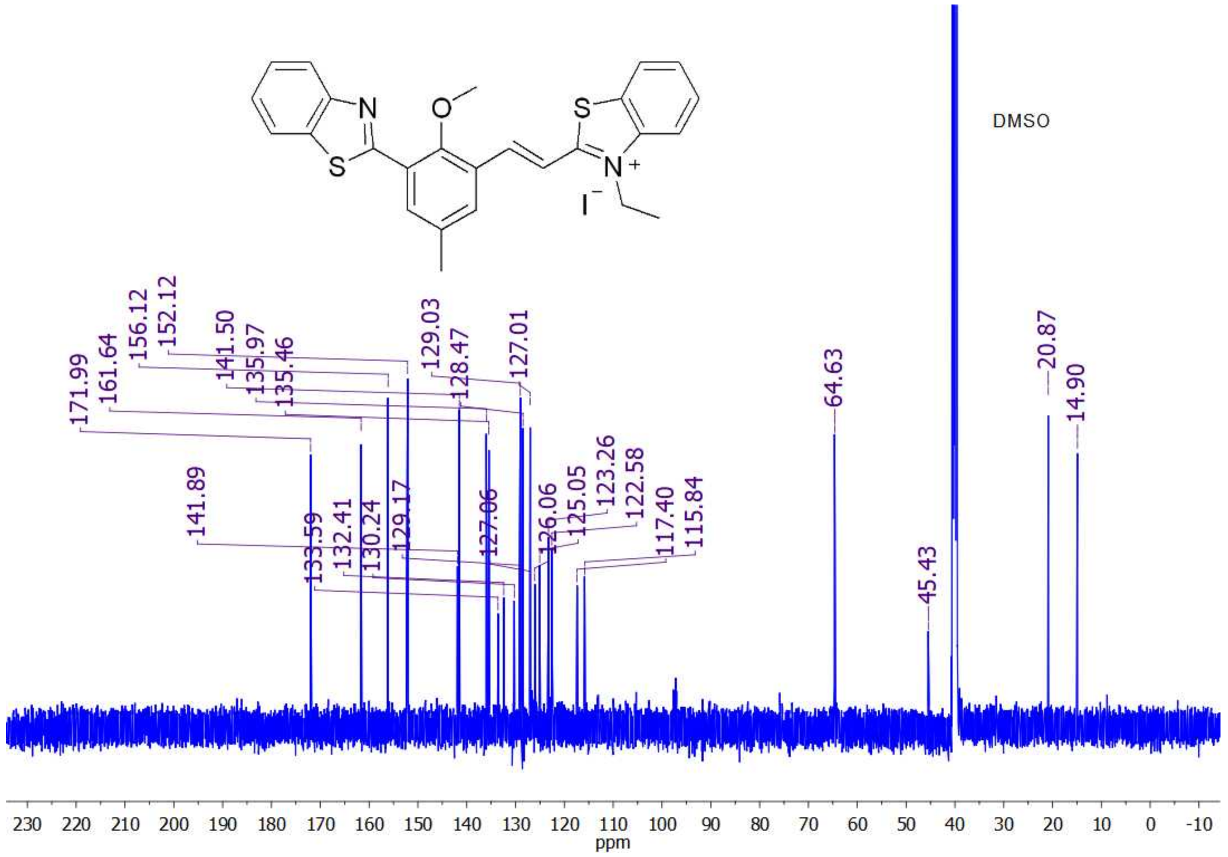

Figure S9. ^{13}C-NMR spectra of compound **6** in DMSO-d$_6$

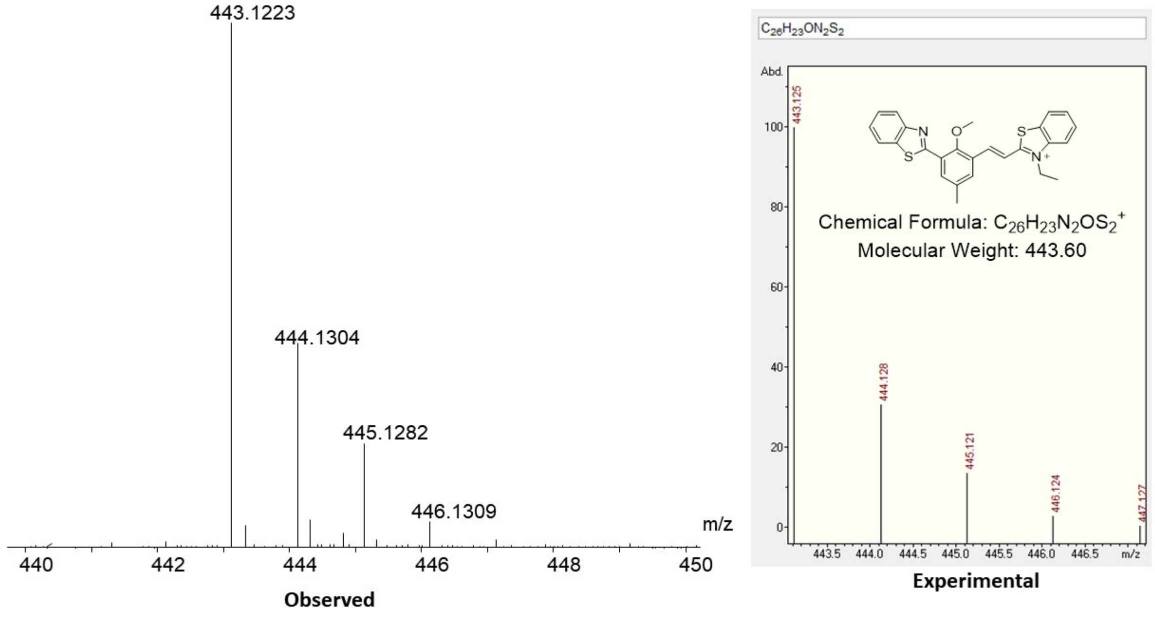

Figure S10. ESI-MS spectra of compound **6**

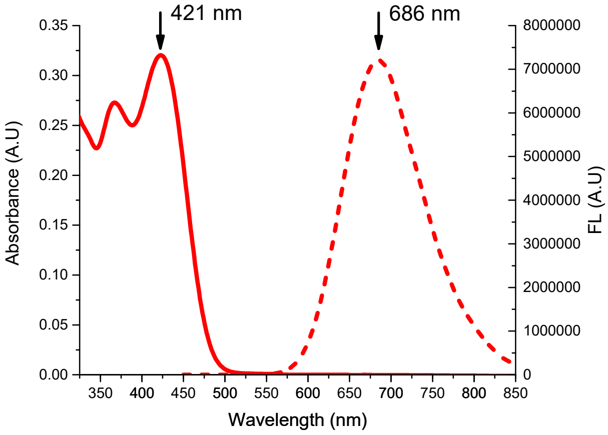

Figure S11. UV-vis absorption (solid line) and emission spectra (broken line) of compound **1** in DCM (10 μM). The excitation wavelength was 430 nm.

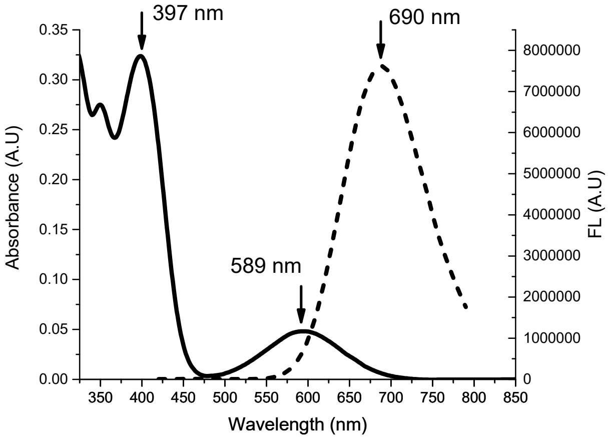

Figure S12. UV-vis absorption (solid line) and emission spectra (broken line) of compound **1** in MeCN (10 μM). The excitation wavelength was 400 nm.

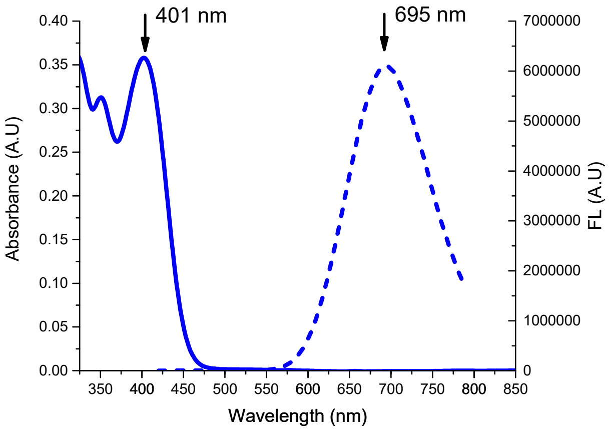

Figure S13. UV-vis absorption (solid line) and emission spectra (broken line) of compound **1** in MeOH (10 μM). The excitation wavelength was 400 nm.

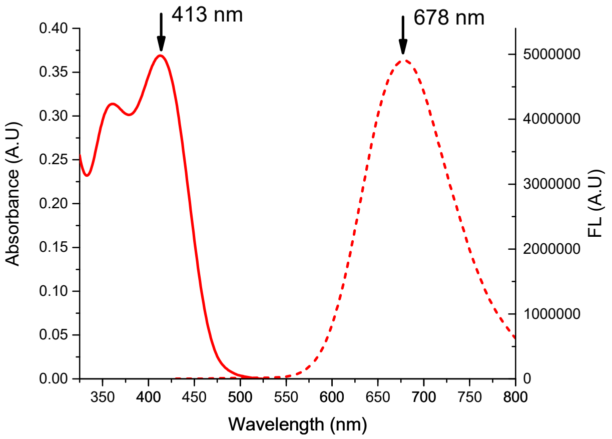

Figure S14. UV-vis absorption (solid line) and emission spectra (broken line) of compound **2** in DCM (10 μM). The excitation wavelength was 410 nm.

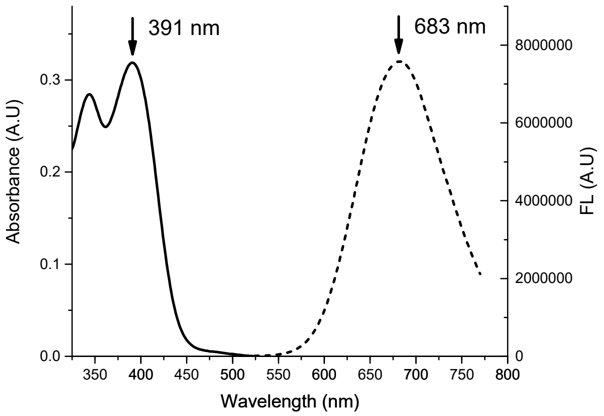

Figure S15. UV-vis absorption (solid line) and emission spectra (broken line) of compound **2** in MeCN (10 μM). The excitation wavelength was 390 nm.

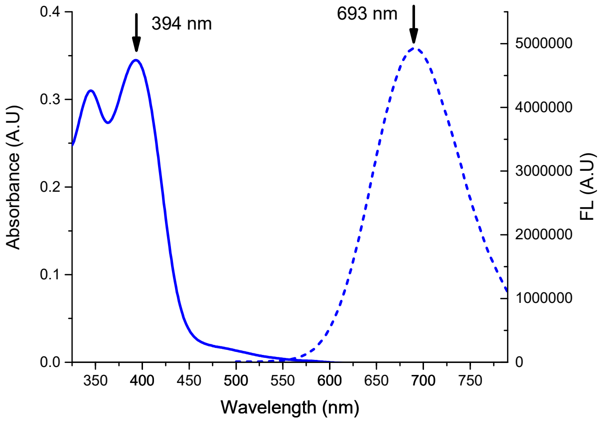

Figure S16. UV-vis absorption (solid line) and emission spectra (broken line) of compound **2** in MeOH (10 μM). The excitation wavelength was 400 nm.

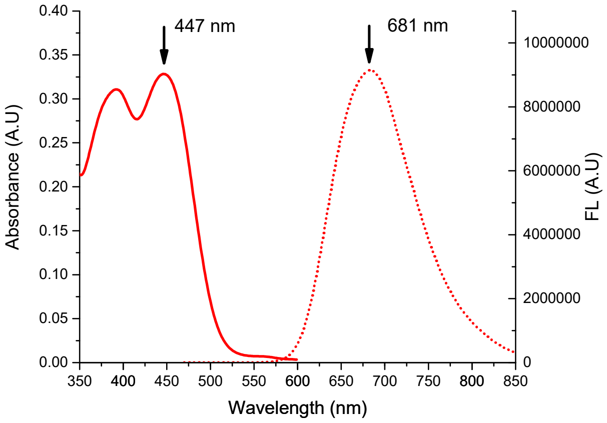

Figure S17. UV-vis absorption (solid line) and emission spectra (broken line) of compound **3** in DCM (10 μM). The excitation wavelength was 450 nm.

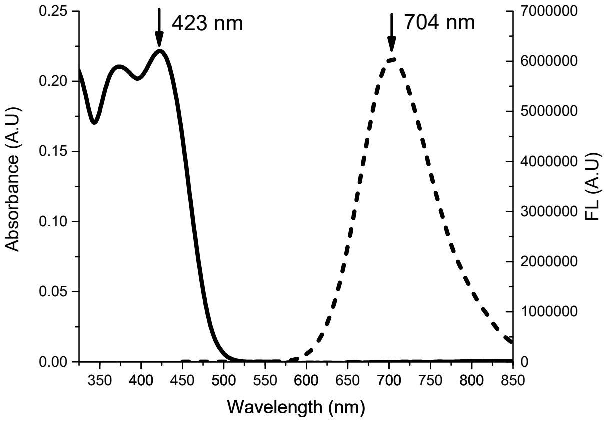

Figure S18. UV-vis absorption (solid line) and emission spectra (broken line) of compound **3** in MeCN (10 μM). The excitation wavelength was 430 nm.

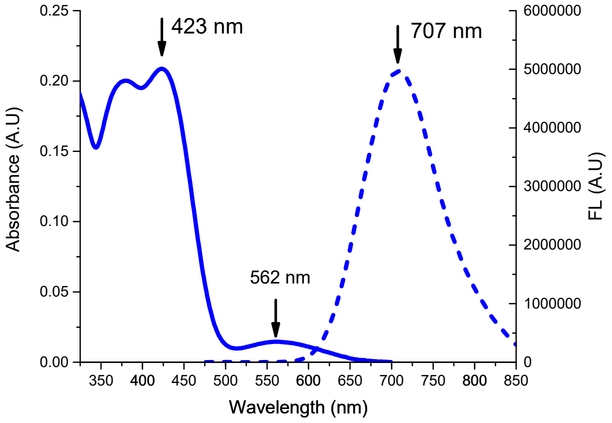

Figure S19. UV-vis absorption (solid line) and emission spectra (broken line) of compound **3** in MeOH (10 μM). The excitation wavelength was 430 nm.

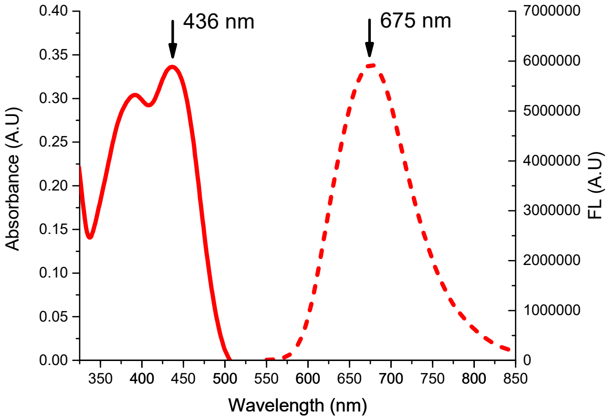

Figure S20. UV-vis absorption (solid line) and emission spectra (broken line) of compound **4** in DCM (10 μM). The excitation wavelength was 440 nm.

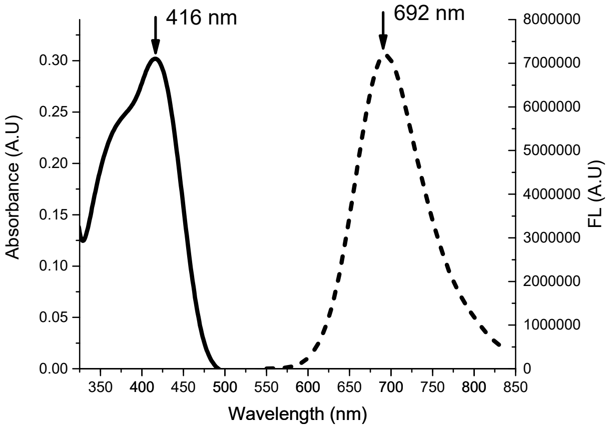

Figure S21. UV-vis absorption (solid line) and emission spectra (broken line) of compound **4** in MeCN (10 μM). The excitation wavelength was 420 nm.

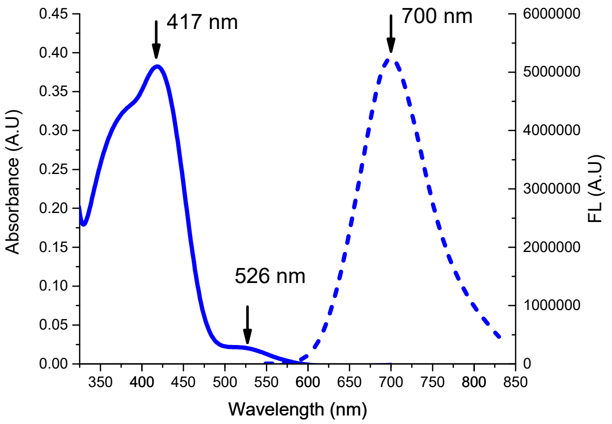

Figure S22. UV-vis absorption (solid line) and emission spectra (broken line) of compound **4** in MeOH (10 μM). The excitation wavelength was 420 nm.

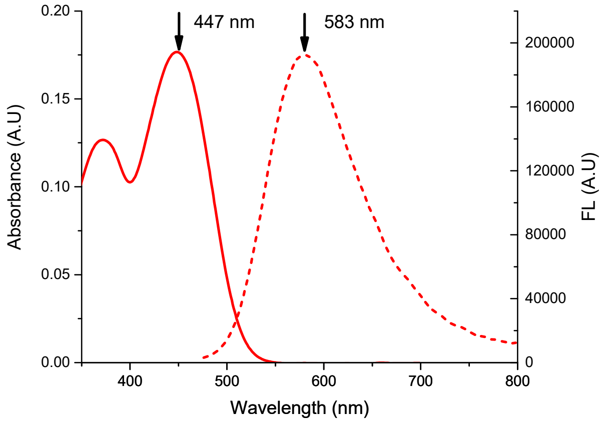

Figure S23. UV-vis absorption (solid line) and emission spectra (broken line) of compound **5** in DCM (10 μM). The excitation wavelength was 450 nm.

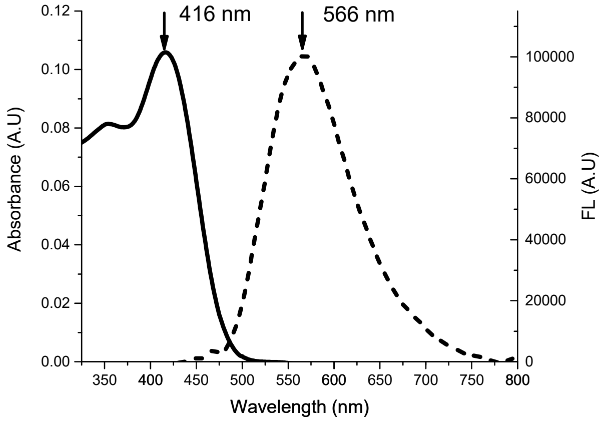

Figure S24. UV-vis absorption (solid line) and emission spectra (broken line) of compound **5** in MeCN (10 μM). The excitation wavelength was 410 nm.

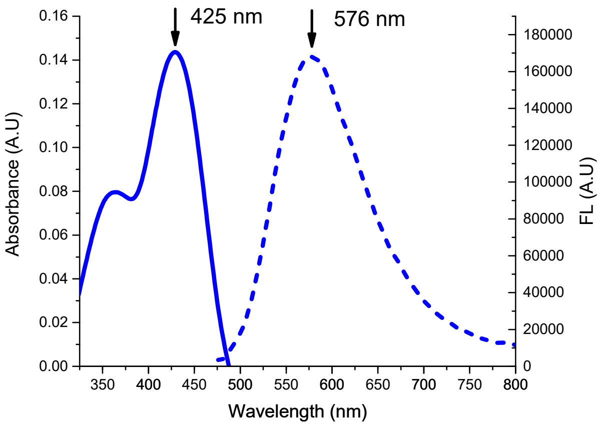

Figure S25. UV-vis absorption (solid line) and emission spectra (broken line) of compound **5** in MeOH (10 μM). The excitation wavelength was 430 nm.

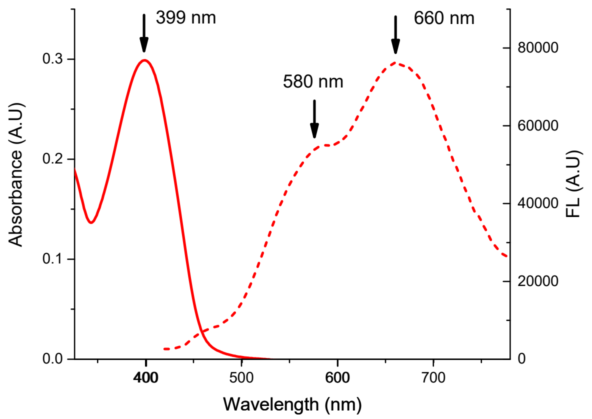

Figure S26. UV-vis absorption (solid line) and emission spectra (broken line) of compound **6** in MeOH (10 μM). The excitation wavelength was 400 nm.

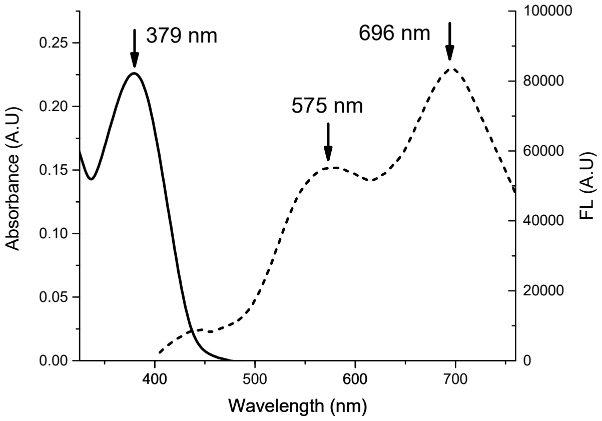

Figure S27. UV-vis absorption (solid line) and emission spectra (broken line) of compound **6** in MeCN (10 μM). The excitation wavelength was 385 nm.

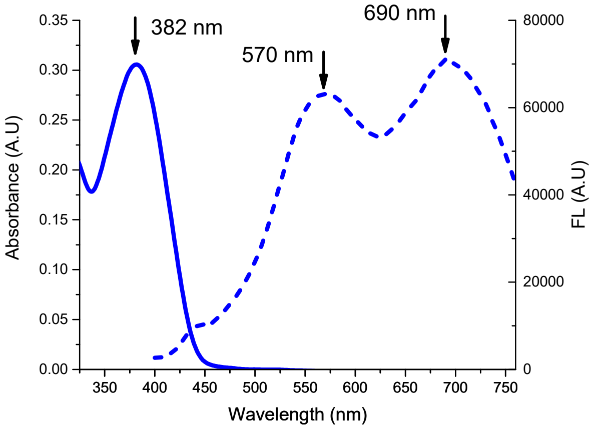

Figure S28. UV-vis absorption (solid line) and emission spectra (broken line) of compound **6** in MeOH (10 μM). The excitation wavelength was 385 nm.

Table S1. Fluorescence lifetime data of compound **1**

**Solvent**	**Lifetime (ns)**		**Normalized pre-exponential**		**Relative amplitude**		**Average lifetime (ns)**	**Chi square (χ^2)**
	τ_1	τ_2	α_1	α_2	f_1	f_2		
**DCM**	1.05	3.38	0.17	0.83	5.84%	94.16%	2.99	1.02
**MeCN**	1.49	2.94	0.17	0.83	9.24%	90.76%	2.69	1.00
**MeOH**	0.90	1.60	0.04	0.96	2.17%	97.83%	1.57	1.30

Table S2. Fluorescence lifetime data of compound **2**

**Solvent**	**Lifetime (ns)**	**Normalized pre-exponential**	**Relative amplitude**		

	τ_1	τ_2	α_1	α_2	f_1	f_2	**Average lifetime (ns)**	**Chi square (χ^2)**
**DCM**	1.27	3.61	0.14	0.86	5.52%	94.48%	3.27	1.03
**MeCN**	1.45	2.91	0.19	0.81	10.43%	89.57%	2.64	1.00
**MeOH**	1.28	1.91	0.68	0.32	58.73%	41.27%	1.48	1.27

Table S3. Fluorescence lifetime data of compound **3**

**Solvent**	**Lifetime (ns)**		**Normalized pre-exponential**		**Relative amplitude**		**Average lifetime (ns)**	**Chi square (χ^2)**
	τ_1	τ_2	α_1	α_2	f_1	f_2		
**DCM**	2.04	2.75	0.20	0.80	15.84%	84.16%	2.60	1.14
**MeCN**	1.29	2.46	0.38	0.62	24.24%	75.76%	2.01	1.09
**MeOH**	1.63	2.20	0.59	0.41	51.22%	48.78%	1.86	1.30

Table S4. Fluorescence lifetime data of compound **4**

**Solvent**	**Lifetime (ns)**		**Normalized pre-exponential**		**Relative amplitude**		**Average lifetime (ns)**	**Chi square (χ^2)**
	τ_1	τ_2	α_1	α_2	f_1	f_2		
**DCM**	1.70	3.07	0.68	0.32	53.97%	46.03%	2.14	1.02
**MeCN**	0.88	2.57	0.40	0.60	18.48%	81.52%	1.90	0.99
**MeOH**	1.22	1.86	0.39	0.61	29.96%	70.04%	1.61	1.18

Table S5. Fluorescence lifetime data of compound **5** (two-exponential)

**Solvent**	**Lifetime (ns)**		**Normalized pre-exponential**		**Relative amplitude**		**Average lifetime (ns)**	**Chi square (χ^2)**
	τ_1	τ_2	α_1	α_2	f_1	f_2		
**DCM**	0.20	1.05	0.99	0.01	97.19%	2.81%	0.21	1.31
**MeCN**	0.13	1.86	1.00	0.00	97.25%	2.75%	0.14	1.26
**MeOH**	0.12	1.08	1.00	0.00	98.94%	1.06%	0.12	1.19

Table S6. Fluorescence lifetime data of compound **5** (three-exponential)

**Solvent**	**Lifetime (ns)**			**Normalized pre-exponential**			**Relative amplitude**			**Average lifetime (ns)**	**Chi square (χ^2)**
	τ_1	τ_2	τ_3	α_1	α_2	α_3	f_1	f_2	f_3		
**DCM**	0.10	0.24	1.77	0.53	0.47	0.00	32.04%	66.62%	1.33%	0.17	1.00
**MeCN**	0.07	0.15	2.04	0.44	0.56	0.00	26.43%	71.03%	2.54%	0.12	1.16
**MeOH**	0.05	0.13	1.25	0.49	0.51	0.00	26.49%	72.69%	0.82%	0.09	1.02

Table S7. Fluorescence lifetime data of compound **6** (550 nm long path filter)

Solvent	Lifetime (ns)			Normalized pre-exponential			Relative amplitude			Average lifetime (ns)	Chi square (χ^2)
	τ_1	τ_2	τ_3	α_1	α_2	α_3	f_1	f_2	f_3		
**DCM**	0.11	1.41	2.61	0.45	0.43	0.12	5.30%	63.24%	31.47%	0.96	1.12
**MeCN**	0.20	0.67	2.45	0.43	0.48	0.09	13.72%	51.03%	35.25%	0.62	1.02
**MeOH**	0.10	0.44	1.59	0.61	0.36	0.03	21.95%	58.18%	19.86%	0.27	1.05
**MEG**	0.05	0.43	1.89	0.74	0.21	0.04	17.31%	43.88%	38.80%	0.21	1.06

Table S8. Fluorescence lifetime data of compound **6** (650 nm long path filter)

Solvent	Lifetime (ns)			Normalized pre-exponential			Relative amplitude			Average lifetime (ns)	Chi square (χ^2)
	τ_1	τ_2	τ_3	α_1	α_2	α_3	f_1	f_2	f_3		
**DCM**	0.10	1.35	2.67	0.27	0.47	0.26	1.99%	46.74%	51.27%	1.35	1.17
**MeCN**	0.18	0.67	2.29	0.33	0.41	0.26	6.22%	29.61%	64.17%	0.93	1.10
**MeOH**	0.05	0.39	1.52	0.62	0.27	0.11	9.47%	34.86%	55.67%	0.30	1.13
**MEG**	0.06	0.35	1.76	0.51	0.33	0.16	6.97%	27.01%	66.03%	0.43	1.16

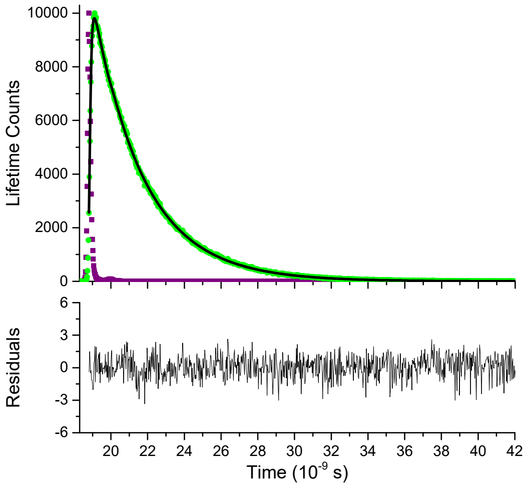

Figure S29. The fluorescence lifetime of IRF (purple square) and decay for compound **1** in MeCN (green circle) and fitted curve of two exponential functions. The residuals are reported in the lower panel. With DD-405L (λ_{em}=406 nm) as light source and 650 nm long path filter.

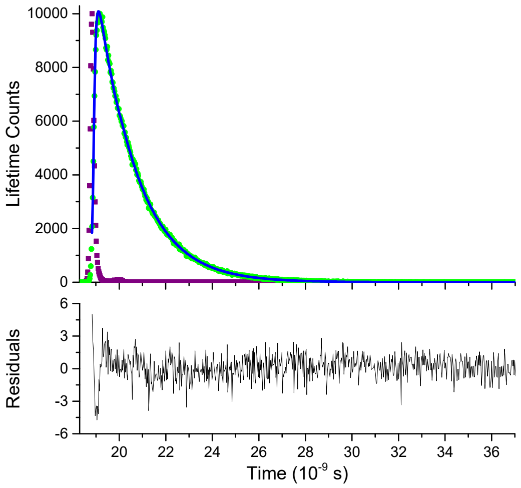

Figure S30. The fluorescence lifetime of IRF (purple square) and decay for compound **1** in MeOH (green circle) and fitted curve of two exponential functions. The residuals are reported in the lower panel. With DD-405L (λ_{em}=406 nm) as light source and 650 nm long path filter.

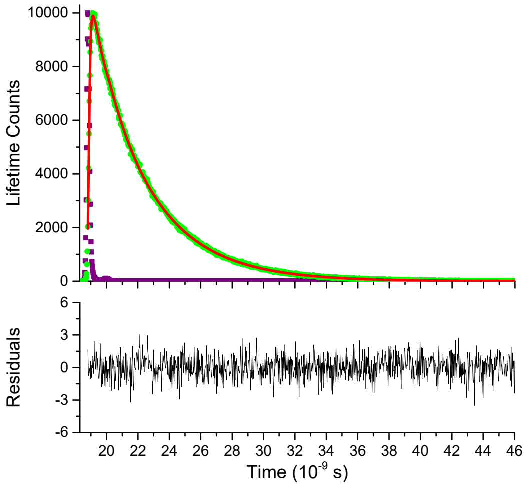

Figure S31. The fluorescence lifetime of IRF (purple square) and decay for compound **2** in DCM (green circle) and fitted curve of two exponential functions. The residuals are reported in the lower panel. With DD-405L (λ_{em}=406 nm) as light source and 650 nm long path filter.

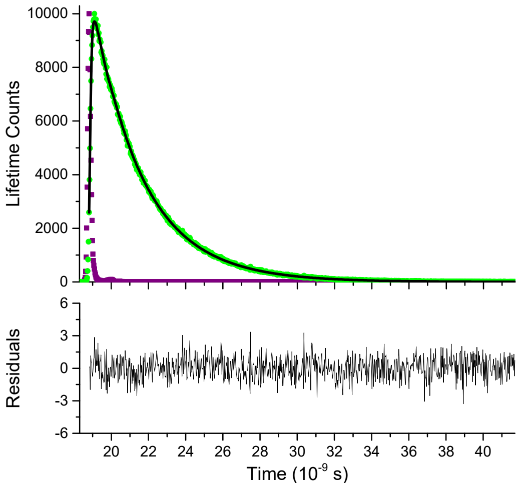

Figure S32. The fluorescence lifetime of IRF (purple square) and decay for compound **2** in MeCN (green circle) and fitted curve of two exponential functions. The residuals are reported in the lower panel. With DD-405L (λ_{em}=406 nm) as light source and 650 nm long path filter.

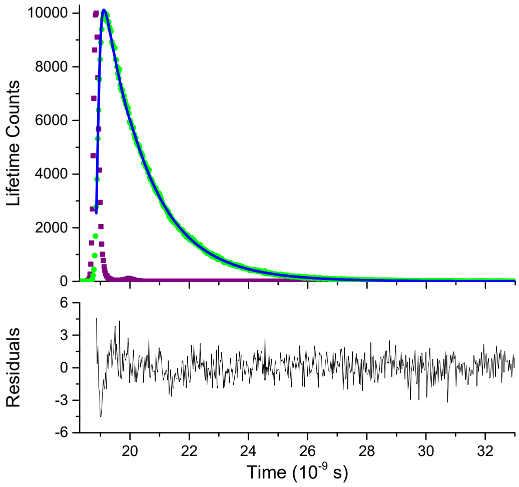

Figure S33. The fluorescence lifetime of IRF (purple square) and decay for compound **2** in MeOH (green circle) and fitted curve of two exponential functions. The residuals are reported in the lower panel. With DD-405L (λ_{em}=406 nm) as light source and 650 nm long path filter.

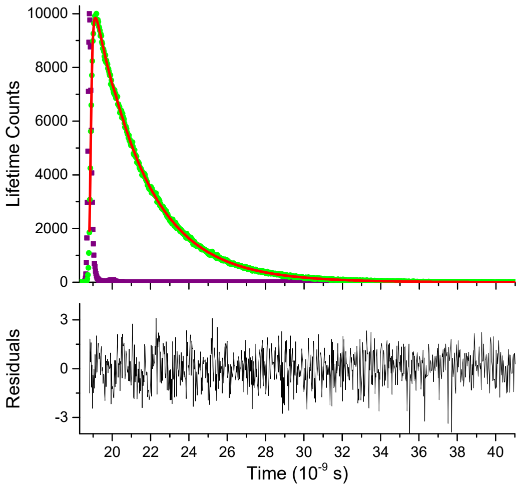

Figure S34. The fluorescence lifetime of IRF (purple square) and decay for compound **3** in DCM (green circle) and fitted curve of two exponential functions. The residuals are reported in the lower panel. With DD-405L (λ_{em}=406 nm) as light source and 650 nm long path filter.

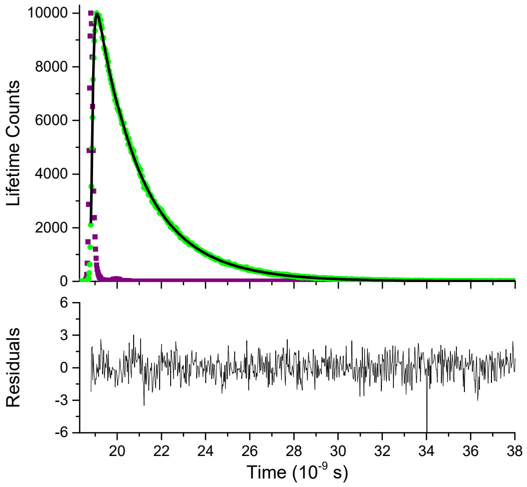

Figure S35. The fluorescence lifetime of IRF (purple square) and decay for compound **3** in MeCN (green circle) and fitted curve of two exponential functions. The residuals are reported in the lower panel. With DD-405L (λ_{em}=406 nm) as light source and 650 nm long path filter.

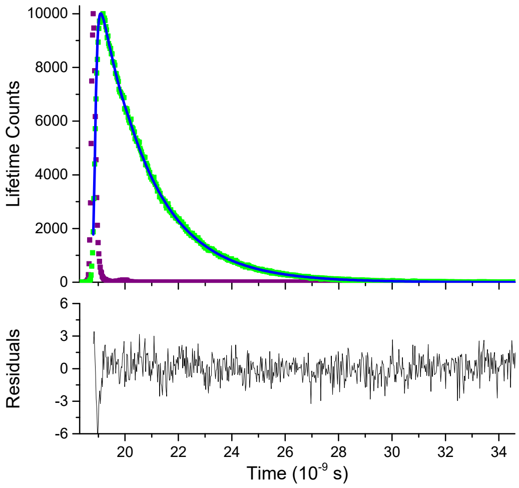

Figure S36. The fluorescence lifetime of IRF (purple square) and decay for compound **3** in MeOH (green circle) and fitted curve of two exponential functions. The residuals are reported in the lower panel. With DD-405L (λ_{em}=406 nm) as light source and 650 nm long path filter.

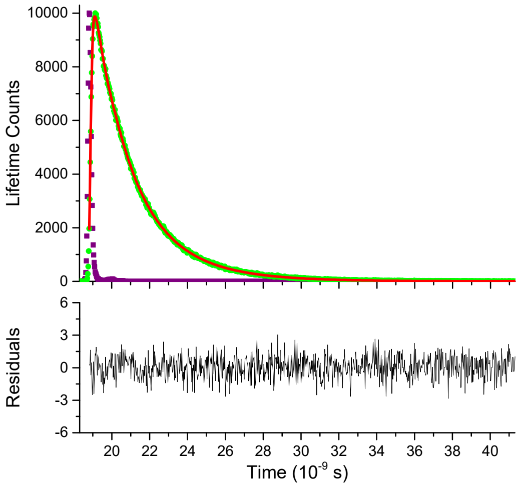

Figure S37. The fluorescence lifetime of IRF (purple square) and decay for compound **4** in DCM (green circle) and fitted curve of two exponential functions. The residuals are reported in the lower panel. With DD-405L (λ_{em}=406 nm) as light source and 650 nm long path filter.

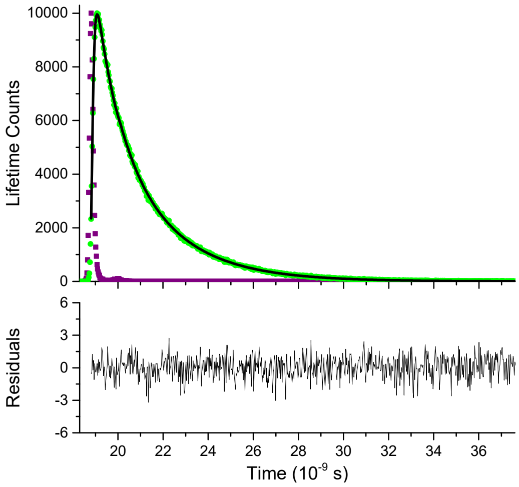

Figure S38. The fluorescence lifetime of IRF (purple square) and decay for compound **4** in MeCN (green circle) and fitted curve of two exponential functions. The residuals are reported in the lower panel. With DD-405L (λ_{em}=406 nm) as light source and 650 nm long path filter.

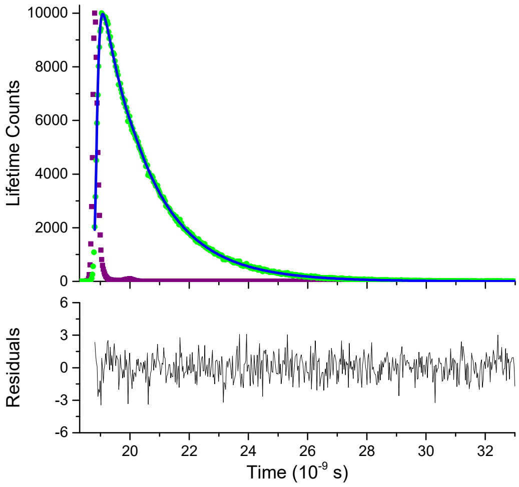

Figure S39. The fluorescence lifetime of IRF (purple square) and decay for compound **4** in MeOH (green circle) and fitted curve of two exponential functions. The residuals are reported in the lower panel. With DD-405L (λ_{em}=406 nm) as light source and 650 nm long path filter.

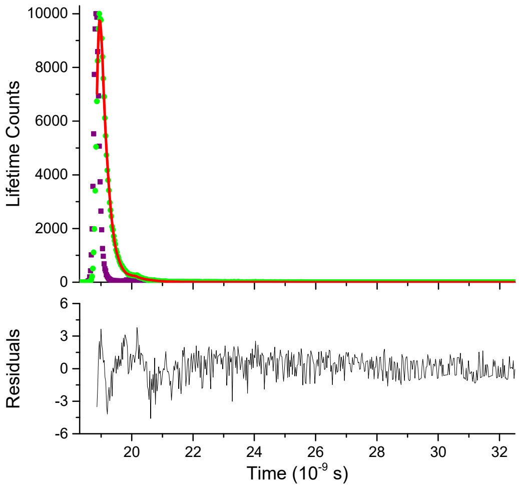

Figure S40. The fluorescence lifetime of IRF (purple square) and decay for compound **5** in DCM (green circle) and fitted curve of two exponential functions. The residuals are reported in the lower panel. With DD-405L (λ_{em}=406 nm) as light source and 550 nm long path filter.

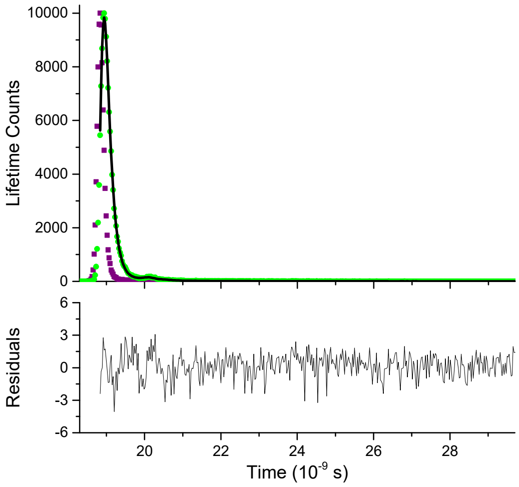

Figure S41. The fluorescence lifetime of IRF (purple square) and decay for compound **5** in MeCN (green circle) and fitted curve of two exponential functions. The residuals are reported in the lower panel. With DD-405L (λ_{em}=406 nm) as light source and 550 nm long path filter.

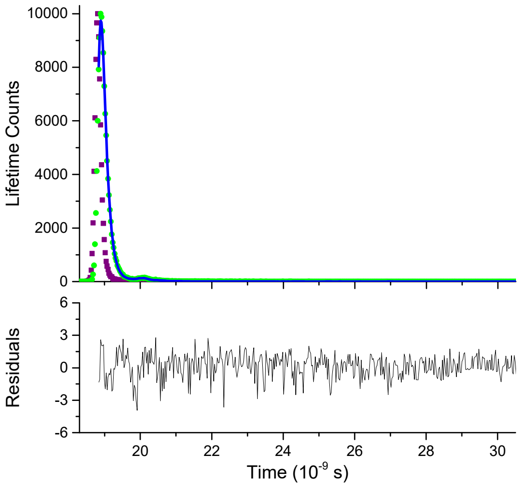

Figure S42. The fluorescence lifetime of IRF (purple square) and decay for compound **5** in MeOH (green circle) and fitted curve of two exponential functions. The residuals are reported in the lower panel. With DD-405L (λ_{em}=406 nm) as light source and 550 nm long path filter.

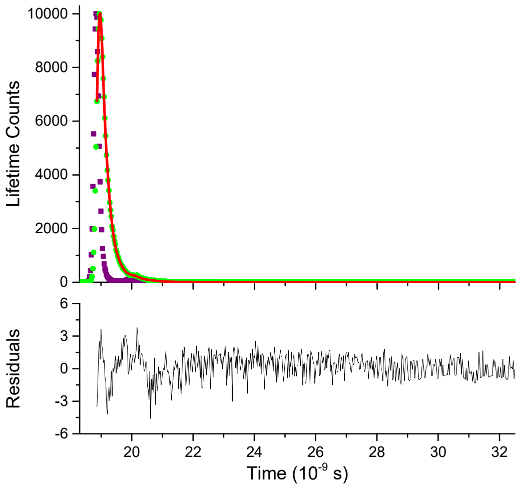

Figure S43. The fluorescence lifetime of IRF (purple square) and decay for compound **5** in DCM (green circle) and fitted curve of three exponential functions. The residuals are reported in the lower panel. With DD-405L (λ_{em}=406 nm) as light source and 550 nm long path filter.

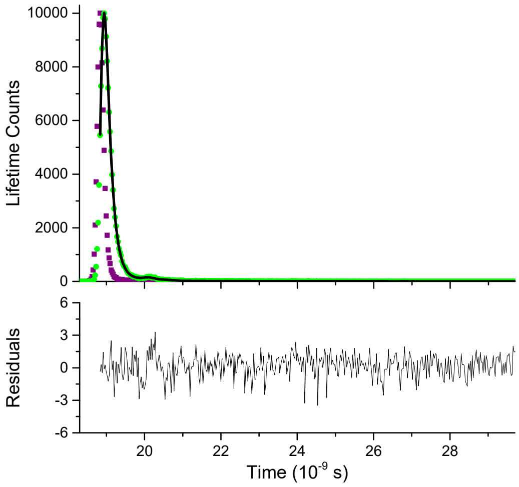

Figure S44. The fluorescence lifetime of IRF (purple square) and decay for compound **5** in MeCN (green circle) and fitted curve of three exponential functions. The residuals are reported in the lower panel. With DD-405L (λ_{em}=406 nm) as light source and 550 nm long path filter.

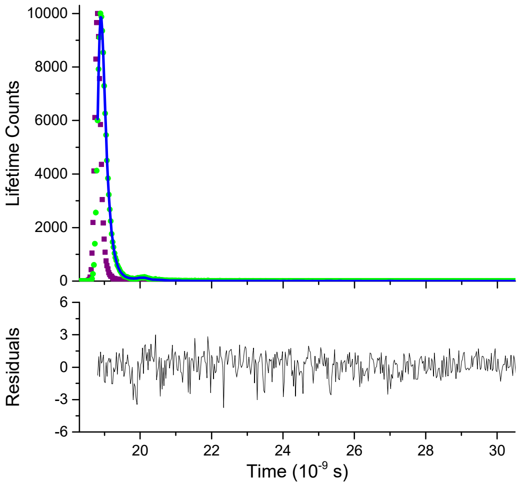

Figure S45. The fluorescence lifetime of IRF (purple square) and decay for compound **5** in MeOH (green circle) and fitted curve of three exponential functions. The residuals are reported in the lower panel. With DD-405L (λ_{em}=406 nm) as light source and 550 nm long path filter.

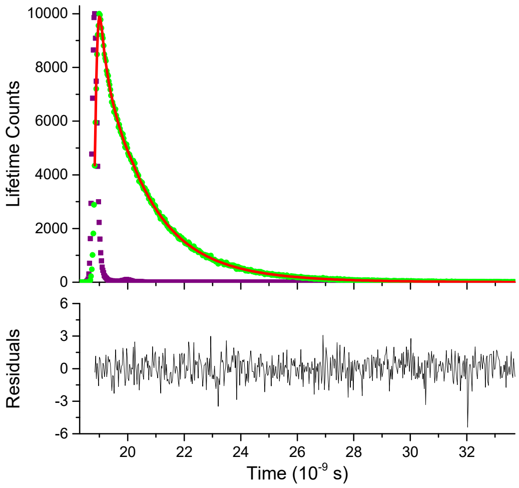

Figure S46. The fluorescence lifetime of IRF (purple square) and decay for compound **6** in DCM (green circle) and fitted curve of three exponential functions. The residuals are reported in the lower panel. With DD-405L (λ_{em}=406 nm) as light source and 550 nm long path filter.

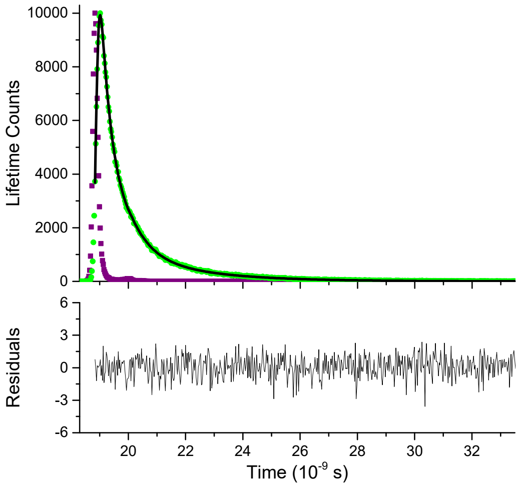

Figure S47. The fluorescence lifetime of IRF (purple square) and decay for compound **6** in MeCN (green circle) and fitted curve of three exponential functions. The residuals are reported in the lower panel. With DD-405L (λ_{em}=406 nm) as light source and 550 nm long path filter.

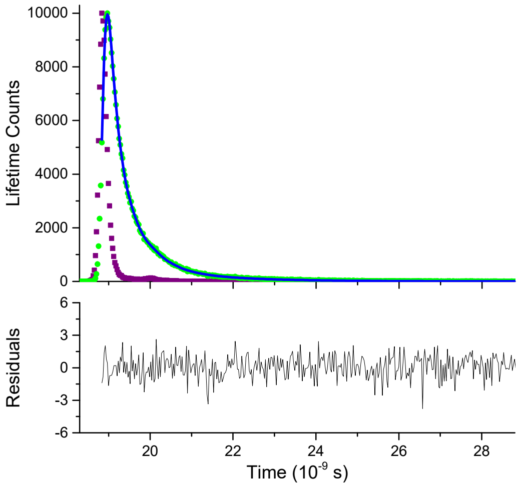

Figure S48. The fluorescence lifetime of IRF (purple square) and decay for compound **6** in MeOH (green circle) and fitted curve of three exponential functions. The residuals are reported in the lower panel. With DD-405L (λ_{em}=406 nm) as light source and 550 nm long path filter.

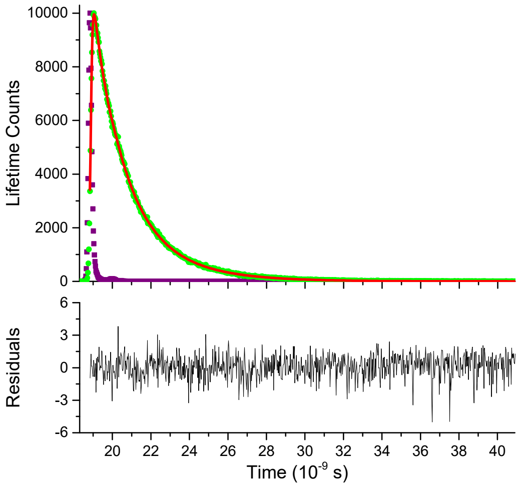

Figure S49. The fluorescence lifetime of IRF (purple square) and decay for compound **6** in DCM (green circle) and fitted curve of three exponential functions. The residuals are reported in the lower panel. With DD-405L (λ_{em}=406 nm) as light source and 650 nm long path filter.

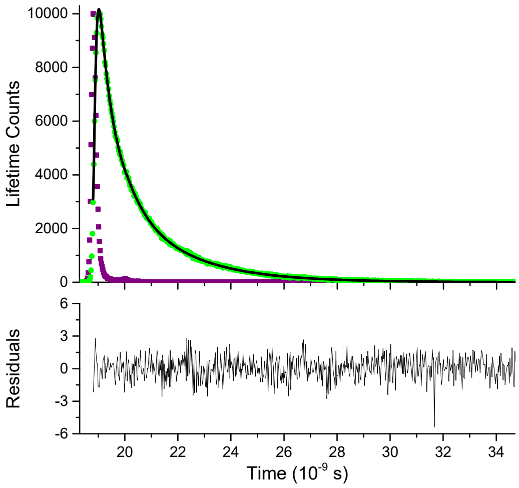

Figure S50. The fluorescence lifetime of IRF (purple square) and decay for compound **6** in MeCN (green circle) and fitted curve of three exponential functions. The residuals are reported in the lower panel. With DD-405L (λ_{em}=406 nm) as light source and 650 nm long path filter.

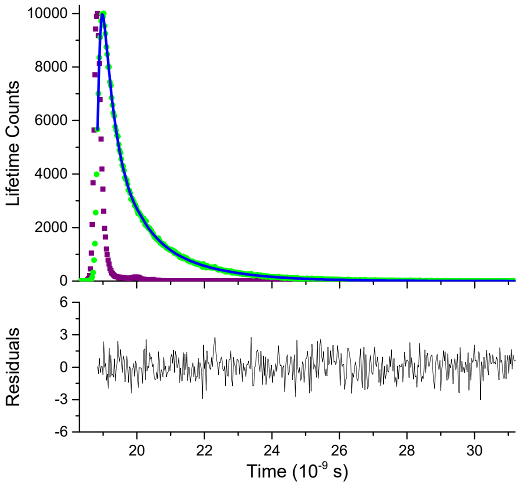

Figure S51. The fluorescence lifetime of IRF (purple square) and decay for compound **6** in MeOH (green circle) and fitted curve of three exponential functions. The residuals are reported in the lower panel. With DD-405L (λ_{em}=406 nm) as light source and 650 nm long path filter.

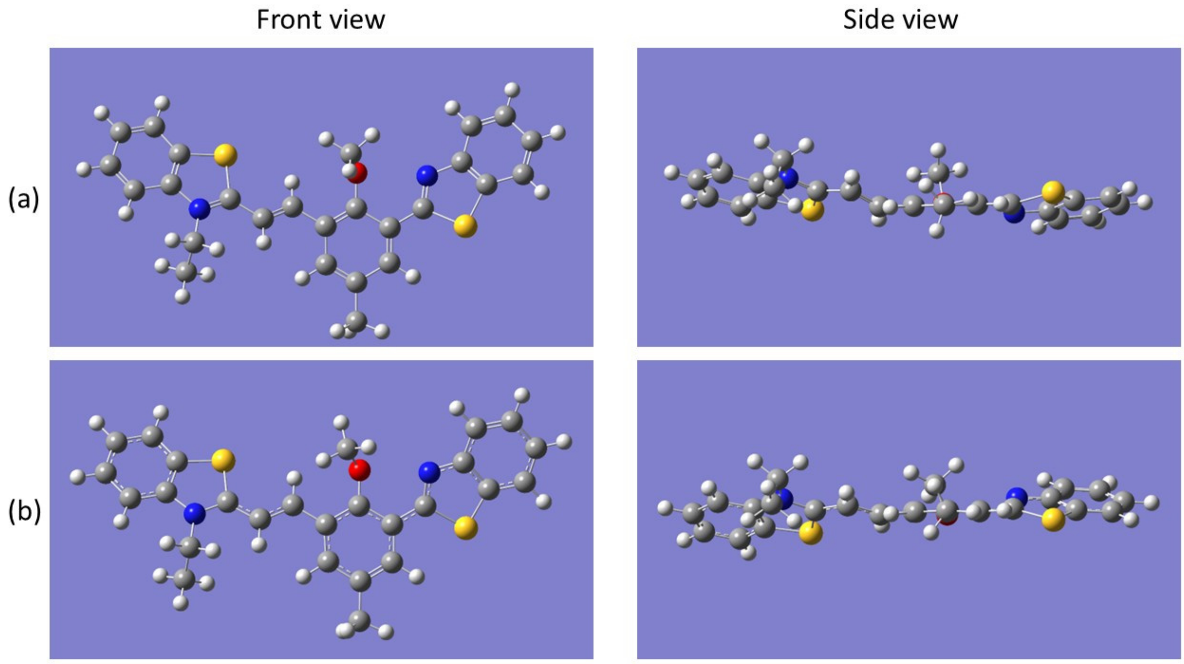

Figure S52. The simulated molecular geometry of compound **6** on the ground state via DFT method (a) and on the excited state via TD-DFT method (b) at B3LYP/6-31G (d, p) level.

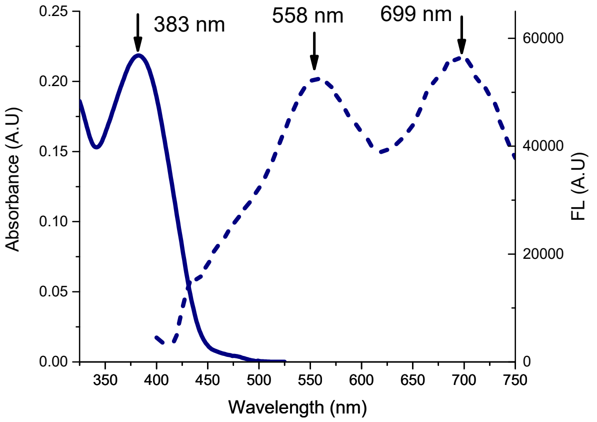

Figure S53. UV-vis absorption (solid line) and emission spectra (broken line) of compound **6** (10 μM) in Ethylene Glycol (MEG). The excitation wavelength was 385 nm.

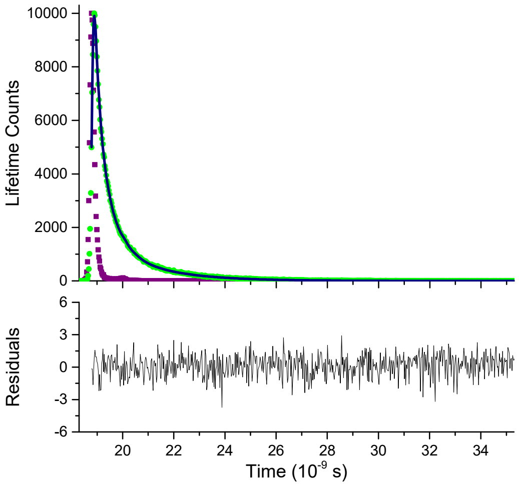

Figure S54. The fluorescence lifetime of IRF (purple square) and decay for compound **6** in MEG (green circle) and fitted curve of three exponential functions. The residuals are reported in the lower panel. With DD-405L (λ_{em}=406 nm) as light source and 550 nm long path filter.

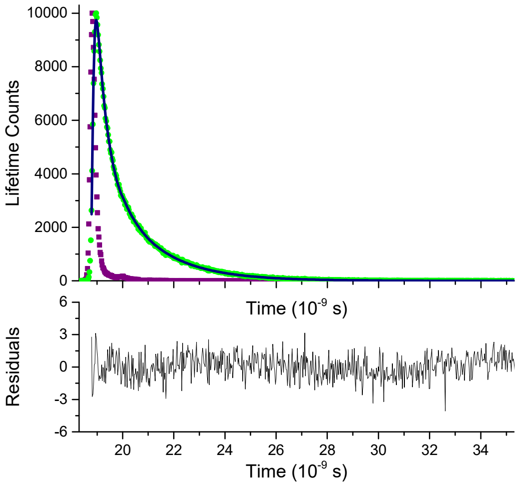

Figure S55. The fluorescence lifetime of IRF (purple square) and decay for compound **6** in MEG (green circle) and fitted curve of three exponential functions. The residuals are reported in the lower panel. With DD-405L (λ_{em}=406 nm) as light source and 650 nm long path filter.
